# MoS_2_/h-BN/Graphene Heterostructure and Plasmonic Effect for Self-Powering Photodetector: A Review

**DOI:** 10.3390/ma14071672

**Published:** 2021-03-29

**Authors:** Umahwathy Sundararaju, Muhammad Aniq Shazni Mohammad Haniff, Pin Jern Ker, P. Susthitha Menon

**Affiliations:** 1 Institute of Microengineering and Nanoelectronics (IMEN), Universiti Kebangsaan Malaysia (UKM), Bangi 43600, Malaysia; p100860@siswa.ukm.edu.my (U.S.); aniqshazni@ukm.edu.my (M.A.S.M.H.); 2 Institute of Sustainable Energy (ISE), Universiti Tenaga Nasional (UNITEN), Kajang 43000, Malaysia; PinJern@uniten.edu.my

**Keywords:** 2D materials, MoS_2_, h-BN, graphene, photodetector, self-powering, plasmonic

## Abstract

A photodetector converts optical signals to detectable electrical signals. Lately, self-powered photodetectors have been widely studied because of their advantages in device miniaturization and low power consumption, which make them preferable in various applications, especially those related to green technology and flexible electronics. Since self-powered photodetectors do not have an external power supply at zero bias, it is important to ensure that the built-in potential in the device produces a sufficiently thick depletion region that efficiently sweeps the carriers across the junction, resulting in detectable electrical signals even at very low-optical power signals. Therefore, two-dimensional (2D) materials are explored as an alternative to silicon-based active regions in the photodetector. In addition, plasmonic effects coupled with self-powered photodetectors will further enhance light absorption and scattering, which contribute to the improvement of the device’s photocurrent generation. Hence, this review focuses on the employment of 2D materials such as graphene and molybdenum disulfide (MoS_2_) with the insertion of hexagonal boron nitride (h-BN) and plasmonic nanoparticles. All these approaches have shown performance improvement of photodetectors for self-powering applications. A comprehensive analysis encompassing 2D material characterization, theoretical and numerical modelling, device physics, fabrication and characterization of photodetectors with graphene/MoS_2_ and graphene/h-BN/MoS_2_ heterostructures with plasmonic effect is presented with potential leads to new research opportunities.

## 1. Introduction

Light detection mechanism was introduced back in 1887 when Heinrich Hertz discovered that electrodes generate electric sparks when illuminated with ultraviolet light. This was supported with Max Planck’s suggestion that energy carried by electromagnetic fields can be quantized as discrete packets, known as photons or quanta, while studying black-body radiation in 1900. Further enlightenment was made possible with Albert Einstein’s explanation of the photoelectric effect with the support of experimental results. The commercial product of photon detection is the photomultiplier tube (PMT), which was invented in the RCA laboratories in 1930. The limitation of PMTs in terms of high sensitivity towards the magnetic field, high cost, and a complicated mechanical structure opened the opportunity for development of new photodetectors, especially devices that employ semiconductor materials.

A device that detects light from the electromagnetic wave spectrum between ultraviolet (0.25–0.4 µm), visible (0.45–0.8 µm) and near infrared (0.9–1.7 µm) and converts it to measurable electrical signals in terms of current, voltage or resistance is known as a photodetector [1,2]. Incident photons are absorbed in the active region by semiconducting material, thus generating electron–hole pairs (EHP) that are collected as photocurrent at the electrodes, which is known as the photogeneration process. The active region of a homojunction P–N photodetector is the depletion region, which is also known as the space charge layer (SCL) that has a built-in potential. The operation of a photodetector can be explained either with or without an external power supply [3,4]. The photogeneration process is enhanced, and the generated carriers are accelerated with the presence of an external power supply in reverse biasing, which improves the photocurrent and performances of the device. On the other hand, the built-in potential in the depletion region will split and drift the photogenerated EHP to produce photocurrent in the photodetector circuit without an external power supply. This is known as the photovoltaic effect in the photodetector biasing, which is the response mechanism of self-powering photodetectors. [5,6]. 

Although photodetectors with external power supply mode have better performance compared to non-biased photodetectors, this increases the size of the device, making it bulky and inconvenient to be packaged. Moreover, the larger device size limits its applications in many fields, especially for remote sensing and wireless applications. Therefore, self-powering photodetectors that utilize the photovoltaic effect have become preferable and have been intensively studied till now. Self-powering photodetectors have many advantages such as small size, light weight, low cost, low power consumption, high photoresponsivity, fast response and most importantly it works independently without an external power supply and relies on the built-in potential, which contributes to energy savings [3,4,7]. However, the photogenerated carriers hinder the absorption of weak optical signals, which requires an additional mechanism to increase the depletion region width and the electric field at the P–N-junction in the self-powered photodetector. Therefore, this requires the exploration of two-dimensional (2D) materials and plasmonic effects to tackle the existing drawbacks in self-powered photodetectors.

**Figure 1:**
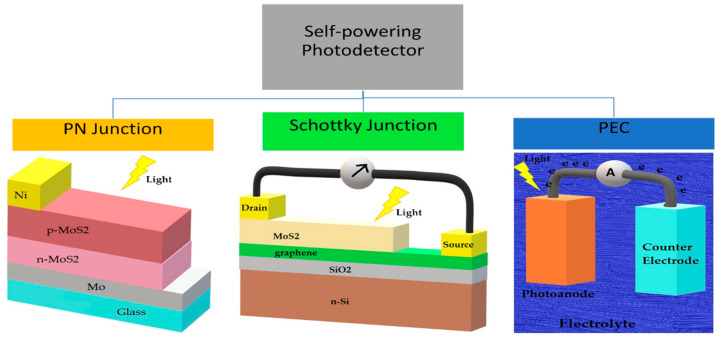
Types of self-powered photodetectors.

In general, there are several types of device configurations for self-powered photodetectors, which are the P–N junction type, Schottky junction type and photoelectrochemical (PEC) type [3,4,7,8]. The separation of photogenerated carriers in the P–N and Schottky junction photodetector types rely on the built-in potential of the active region materials, whereas the PEC type photodetector performance is dependent on the energy barrier between the electrode materials and electrolyte. In this case, the response mechanism of self-powered photodetectors does not utilize an external biasing and is totally dependent on the light source that illuminates the device. The device schematics for all these three types of self-powered photodetectors are shown in Figure 1.

The P–N junction type self-powered photodetector has more advantages compared to other types of photodetectors due to its simple structure, low dark current, high sensitivity, high stability and fast response time. Although the homojunction P–N photodetector achieved great progress in the past, it has performance limitations in terms of weak absorption of photons due to the limited material bandgap energy, especially in self-powering photodetectors. This limitation can be resolved in the heterojunction P–N photodetector, which has two or more materials with different bandgaps that promotes light absorption. Nevertheless, designing heterostructures is challenging, as it is a trade-off parameter towards the performance of the photodetector because recombination may occur at the heterostructure interface. The Schottky junction type has better response speed and higher sensitivity and relies on the presence of a built-in potential, which is in contrast with the ohmic contact photodetector, although it has a large photoconductive gain and higher responsivity. Schottky junction photodetectors can be classified into semiconductor–metal Schottky junction and semiconductor–graphene Schottky junction. The latter has demonstrated outstanding performance, and further enhancement was achievable through interface engineering. PEC is known as a new type of self-powering photodetector with a simpler manufacturing process and larger photoresponsivity but is limited by the stability of the device [3,4].

The venture of employing 2D materials began back in 2004 when graphene was introduced by Geim and colleagues. Ever since then, there have been many emerging 2D materials that were employed specially for photodetection applications ranging from graphene, transition metal dichalcogenides (TMDs), 2D perovskites, 2D metal–organic frameworks (MOFs) and covalent organic frameworks (COFs), and graphdiynes. Graphene is well known for its unique electrical and optical properties and is still actively utilized in photodetectors. Since the gapless nature of graphene limits its optical performances in photodetectors, it is now being hybridized with other semiconducting materials, especially from the family of 2D materials, and acts as the charge transport layer in the device. In the context of TMDs, there are many famously explored materials such as MoS_2_, WS_2_ and WSe_2_.

The performances of photodetectors determine their relevance of applications in various fields. The improved photoresponse of 2D material-based photodetectors makes them useful for application in areas such as military and civil areas [9], digital and medical imaging [10,11], optical communication [11,12], night vision [11], environmental monitoring as well as wearable electronics [3,11]. For example, a flexible and wearable photodetector was realized using a few layers of MoS_2_ as a large area flexible photodetector with photoresponse of approximately 20 mA/W with response time of a few seconds [9].

Although MoS_2_-based photodetectors have demonstrated a lower performance compared to other counterparts, a significant improvement in terms of photoresponsivity was shown when MoS_2_ was hybridized with graphene. Adding to this, further improvements were shown by inserting the hexagonal boron nitride (h-BN) in forming the metal–insulator–semiconductor (MIS) structures, which contributed significantly in terms of lowering the dark current in photodetectors. To the best of the authors’ knowledge, the study on optical devices that employ graphene/h-BN/MoS_2_ heterostructures was developed in 2015 [13] and is being continued till the present. Many device performances were reported in terms of improved responsivity, specific detectivity and higher I_on_/I_off_ ratios where the breakthrough highlight of restoring the photovoltaic effect with van der Waals heterojunctions for self-powered photodetectors was discovered in 2019. The h-BN insertion between graphene and MoS_2_ showed three orders of improvement in photocurrent and specific detectivity of 6.7 × 10^10^ Jones with external quantum efficiency of over 80% at zero bias. Since the graphene/h-BN/MoS_2_ heterostructures have potential in self-powering photodetectors, further studies must be continued to tailor the device structures and modify the behavior of these materials for optimal performance, which is crucial for self-powered photodetectors because these advanced devices are expected to detect the smallest light signal without relying on external biasing. Therefore, it is important to comprehend and summarize the device physics, fabrication process, working principles and advances based on previous works on photodetectors that employ graphene, MoS_2_ and h-BN heterostructures to analyze the gap for further improvement. This is the motivation and rational for focusing this review on only graphene, MoS_2_ and h-BN materials.

Generally, there are many reviews related to photodetectors, especially related to self-powering effects such as self-powering nanoscale photodetectors in 2017 [3] and self-powering photodetectors based on 2D materials in 2019 [4]. These reviews indicated that the self-powering photodetectors are being intensively explored and are still relevant to our present study, especially by venturing into the types of materials and structures of the device, which is supported by a review that emphasizes the design approaches for 2D material photodetectors [14]. However, the design approach that is discussed in this review [14] is not merely focusing on self-powering photodetectors. Basically, the performances of self-powered photodetectors are being evaluated by substituting the active and/or charge transport layers with 2D materials, as reported in [4] by considering all the three types of self-powered photodetectors, as mentioned earlier [3]. In the context of materials, graphene-based [6,15] and MoS_2_-based [11] light sensing devices have been extensively reviewed. Previous works focused mainly on the implementation of these materials individually, and with their heterostructures in the devices coupled with other counterpart materials. Some attention was given to graphene/MoS_2_ heterostructures with/without incorporation of other materials [13,16,17,18,19]. However, these papers lacked a thorough review on the effect of the h-BN insertion layer as well as methods to reduce the dark current and enhance photon absorption via the plasmonic effect, which modifies the interaction between light and the materials. The latest review published in 2021 on graphene/MoS_2_ nanohybrid by Choi and team supports the relevance and importance of this review manuscript. They emphasized the potential of synergizing graphene and MoS_2_ for developing various types of biosensors with higher sensitivity for the biomedical field [20]. In contrast, we are aiming to study the properties and performances of photodetectors with graphene and MoS_2_, as well as the insertion of h-BN layers, which is a promising tunnelling layer between graphene and the MoS_2_ heterostructure, based on previous works, to improve the photovoltaics of the device that contribute to the self-powering effect.

Therefore, this review is scoped specifically on the properties and characteristics of graphene/MoS_2_-based heterostructures with/without the insertion of h-BN layers and their relevance for self-powering photodetection applications. Furthermore, the effect of plasmonic coupling with 2D materials towards the performances of photodetection in devices such as photodetectors and solar cells is also reviewed. Although this review emphasizes photodetectors, other devices such as transistors and solar cells with graphene/MoS_2_ or graphene/h-BN/MoS_2_ heterostructures are also included to understand the behaviors and physics of these heterostructures, because this review aims to study the impact of material structuring and attribution of plasmonic effects towards the electrical and optical properties, as well as the photovoltaic effect on graphene/MoS_2_-based devices as a path for self-powering application. Since the majority of the reviewed devices are vertically structured, dimensions are a critical parameter, which is also highlighted in this review. The layout of this review is as such: parameters of photodetectors, 2D materials in photodetectors, plasmonic implementation in photodetectors, numerical modelling for MoS_2_/h-BN/graphene photodetector and the outlook of this study for the future.

## 2. Parameters of Photodetectors

In general, the performance of a photodetector is based on some of the following key parameters, as shown in Table 1 [2,3,4,6,11]. The description, equations and units of these parameters are also listed accordingly.

## 3. Two-Dimensional Materials in Photodetector

Currently, the photodetector market is dominated by silicon-based photodiodes due to the matured fabrication process at low cost. Although silicon is an excellent and abundant semiconductor material, the narrow and indirect bandgap properties of silicon limit the detection spectrum, especially in the near-infrared region, which triggers the search for alternative materials, especially for optoelectronic applications [2]. Materials with wider bandgap such as ZnO, SiC and diamond are considered as better material choices due to advantages such as strong radiation hardness, good thermal and chemical stability as well as feasibility for harsh environmental applications. All these inorganic materials have high carrier mobility and excellent absorption coefficients but are restricted by high temperature synthesis, complex device fabrication and low flexibility. Those drawbacks were resolved by using organic materials but were limited by low carrier mobility. Therefore, hybridization between organic and inorganic material was proposed as a structure for photodetector and is being utilized to date [2,7]. The hybrid structure introduces heterojunctions in photodetectors that lead to built-in potential that can be used for self-powering application.

**Figure 2:**
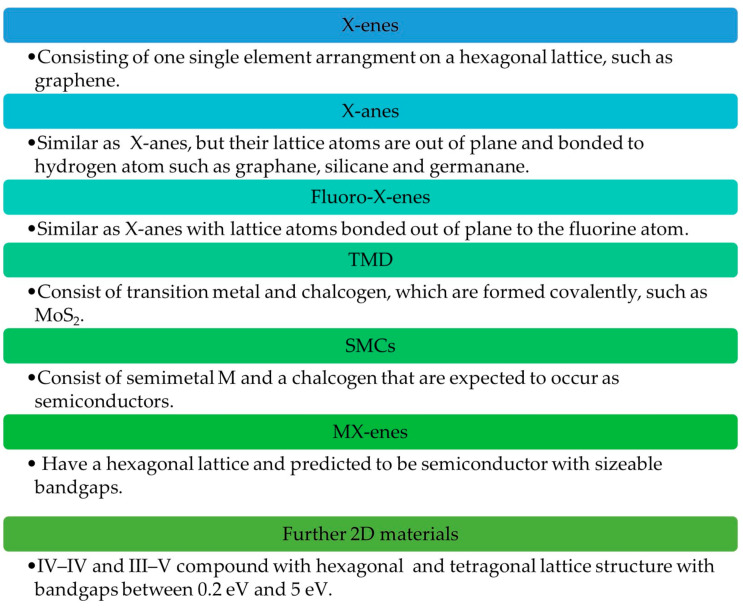
Classification of 2D materials (adapted from [21]).

**Figure 3:**
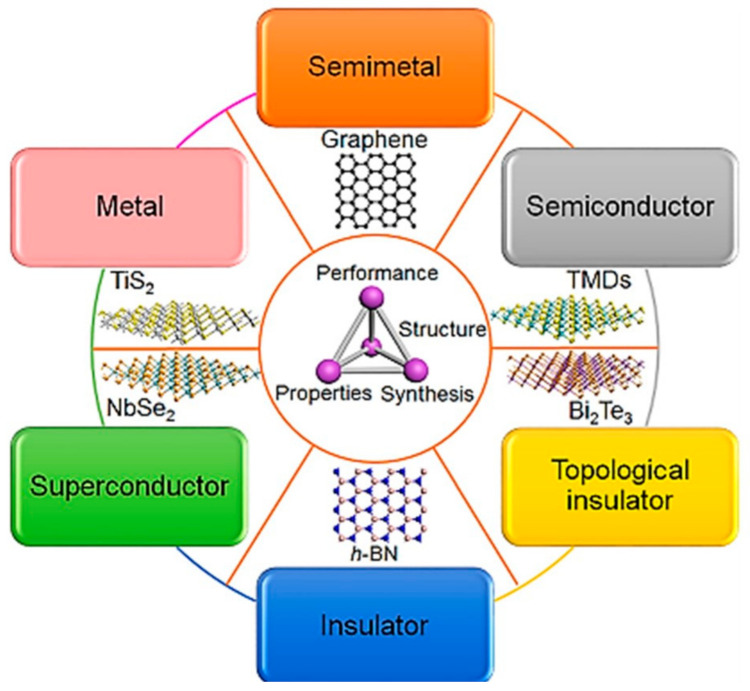
2D material family (Reprinted from [24], with the permission of AIP Publishing).

Photodetectors that are developed using non-2D materials only detect light at the ultraviolet spectrum, which limits their applications and development. Therefore, 2D materials are being extensively explored to push the performance limit further [4]. Intense exploration and examination in preparing stable 2D materials upon successful preparation of graphene led to the discovery of a 140 various different 2D materials with electronic properties from metallic to insulating [21,22,23]. The classes of 2D materials are X-enes, X-anes, Fluro-X-enes, transition metal dichalcogenides (TMDs), SMCs, MX-enes and other 2D materials, which are shown in Figure 2. Some examples of materials from the 2D materials family [21,24] are also illustrated in Figure 3.

Generally, 2D materials have great potential for high-performance photodetectors due to their high crystal quality and unique properties in both electronic and optical aspects such as tunable bandgaps with thickness variations [25], as well as strong covalent bonding between molecular layers with relatively low van Der Waals (vdW) at the interlayer [26]. Generally, 2D materials have large surface-to-volume ratios [19] with ultra-thin lattice structures [27] and flexible integrability [28]. Moreover, 2D materials are sensitive to light, heat and ambient, which make them suitable for broader spectrum detection from the infrared to ultraviolet regime [4,11,27]. Specific crystal structure and atomic layer stacking sequence, especially in TMDs, can vary the phases of 2D materials from the range of metallic, semiconducting, superconducting and insulating [29]. Nevertheless, 2D materials also have their limitations in terms of stability, speed, sensitivity, spectral selectivity and signal-to-noise ratio (SNR) [25]. Therefore, the requirement to meet these criteria were the motivation for studies related to improving the performances of 2D material-based photodetectors till present.

As mentioned earlier, there is a list of 2D materials that are being intensively studied for photodetection applications. However, this review highlights three main materials, which are graphene, MoS_2_ and h-BN with their heterostructures in terms of physics and electronic and optical properties. The integration of excellent electrical properties of graphene, especially in terms of ultra-fast carrier mobility and excellent optical properties of MoS_2_, in terms of large absorption coefficient and gain have been demonstrating significant enhancement in the performance of photodetectors. This is because the beneficial properties from both the materials were combined and the limitations of individual material were tackled by each other, which could contribute towards device performance. Despite intense research on these 2D materials in the past five years, the studies related to photodetectors with graphene and MoS_2_ heterostructures with/without h-BN were still being reported in 2020. The latest studies showed ultrahigh responsivity and detectivity via channel length reduction and Schottky barrier modulation [19], as well as impact of defect states towards photoresponse in graphene/h-BN/MoS_2_ photodetectors [30]. Although both the works did not specifically highlight the self-powering effect, their findings in I–V characteristics showed the potential of their approaches in self-powering application, where dark currents were suppressed and photocurrents were improved at zero biasing or low biasing approaching zero. The continuous exploration of device performance pertaining to graphene–MoS_2_ photodetectors in recent works is the motivation and rationale for selecting these materials in this review, as there is room for further investigation in terms of their device physics and fabrication approaches, which can result in improved photodetectors, especially with self-powering mechanisms.

This section covers briefly the properties of graphene, MoS_2_ and h-BN and focuses on the graphene/h-BN/MoS_2_ heterostructures in terms of device physics and working principles and device fabrication and highlights previous works on the performance of photodetectors with these heterostructures.

### 3.1. Graphene

Graphene is an sp^2^-hybridized carbon atom in a honeycomb lattice structure [31] that has a monoatomic layer thickness, high carrier mobility [6,27,29,32,33], broadband absorption from visible to infrared regime [6,27,32,33,34], high thermal conductivity [29], ultra-fast performance [12,34], high mechanical strength [29] and good toughness. It also has a gate tunability effect [6,33] and is a good candidate for high-frequency optoelectronics [27]. Graphene serves as a transparent electrode with high optical transmittance, large electrical conductivity and tunable work function. Monolayer graphene has 97.7% transparency, which decreases with increases in the number of layers at the visible light spectrum, and 2.3% white light absorption [4,11,29,35]. This limits graphene’s application in photodetectors in the pristine state [36]. Graphene behaves as a Schottky junction when combined with semiconductor materials for self-driven light detections [3,4,37]. The chemically-inert graphene is ideal as a contact electrode in the absence of diffusion and reaction with semiconductor crystals. It also has a tunable Fermi level to lower the contact resistance, which is a dominating problem in photodetectors, or even to create a barrier-free contact with semiconductors due to the finite density of the state of graphene [25].

**Figure 4:**
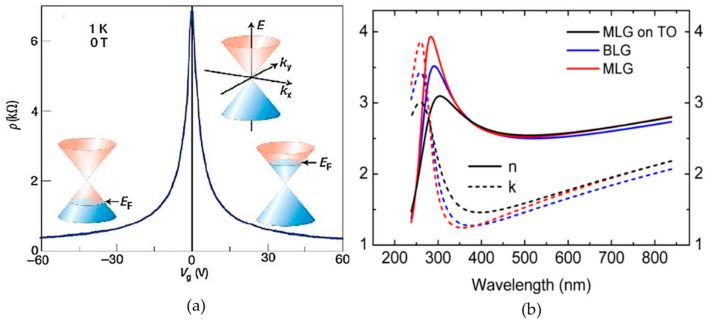
(**a**) Modulation of Fermi level with respect to gate biasing in single layer graphene. The inset shows a schematic of the band structure of pristine graphene with the presence of the Fermi level at the Dirac point (Reprinted from [31], with permission from Elsevier, 2010). (**b**) Refractive index and extinction coefficient for monolayer (red), bilayer (blue) and monolayer graphene with thermal oxide (black) (Republished with the permission of Royal Society of Chemistry, from [39]; permission conveyed through Copyright Clearance Center, Inc. (Danvers, MA, USA), 2015).

Although graphene shows excellent properties for light detecting applications, it is limited by low photoresponsivity, detectivity and quantum efficiency due to its low light absorption coefficient and fast recombination rate [4] with an ultrashort photocarrier lifetime [38]. These drawbacks are due to the absence of a bandgap in graphene that makes it semi-metallic [6,27,29]. Many approaches were explored to tackle this issue, such as graphene bandgap opening with doping method, material engineering to enhance interaction between graphene and device structure engineering [14]. Moreover, the photogating approach can modulate the Fermi level in graphene, which can alter the behavior of graphene to be prone to either p-type or n-type when the Fermi level shifted either below or above the Dirac point, respectively. This will directly modulate the work function of pristine monolayer graphene, which is 4.5 eV. The modulation of the work function of graphene is also dependable to the interaction of graphene with its counterpart material in the device. Graphene was found highly p-doped, which resulted in a work function of 4.68 eV with Fermi level shifted 0.68 eV below the Dirac point, which could be contributed by the underlying substrate, adsorbates and polymer residue, as reported by Rathi et al. [13]. On the other hand, graphene in the self-powered photodetector that was studied by Li et al. showed an n-type behavior with Fermi level shifted 0.17 eV above the Dirac point, which was probably due to electron doping from multilayer MoS_2_ [10]. The tuneability of Fermi level in single layer graphene with respect to gate biasing and the effect towards resistivity is shown in Figure 4a. The presence of Fermi level at the Dirac point in a pristine graphene layer is shown in the inset of Figure 4a. Resistivity in the graphene layer is controlled based on the modulation of Fermi level, which is influenced by the gate biasing. The Fermi level is either lowered or raised from the Dirac point based on negative or positive gate biasing, which denotes the changes in behavior of graphene to be p-type and n-type, respectively. This reveals the ambipolar electric field effect in single layer graphene. The physics of Fermi level shifting is crucial, because it determines the carrier transport of the material, especially at the interface. This will be further discussed in Section 3.4.1.

Other electrical properties of graphene are also reviewed in this section. The intrinsic carrier concentration of graphene at room temperature is $6\times 10^{10}$ cm^−2^ with the carrier concentration of the electron and hole that can be calculated based on the linear Dirac-like dispersion relation, as in Equation (1) [40]. The Fermi velocity is given by $\nu_{F}=1.1\times 10^{6}$ m/s [18]. The carrier mobility, which is a superior property of graphene compared to other materials, was reported at an ultra-high value of 200,000 cm^2^/Vs for the mechanically exfoliated suspended layer [41]. However, the carrier mobility was reported to be 15,000 cm^2^/Vs for a graphene layer on silicon dioxide with a stable lattice structure at room temperature [4,42] due to the scattering effect. The carrier mobility of a graphene layer on a silicon dioxide substrate was recorded up to 40,000 cm^2^/Vs compared to a hexagonal boron nitride substrate, which reported carrier mobility up to 100,000 cm^2^/Vs [41]. This clearly shows that the carrier mobility of graphene also depends on the type of substrate.

(1) $$N_{e/h}=\frac{{\left(\Delta E_{D}\right)}^{2}}{\pi {\left(\hbar \nu_{F}\right)}^{2}}$$

Besides electrical properties, optical properties of graphene were also studied in previous works [39,43]. The refractive index (n) and extinction coefficient (k) were evaluated for CVD-grown graphene. The n and k values for monolayer graphene, bilayer graphene and monolayer graphene with thermal oxide are shown in Figure 4 based on the work in [39]. The parameter of n determines the amount of light that is refracted when entering a material, while k indicates the amount of attenuation when light propagates through the material. On the other hand, the complex refractive index of mechanically exfoliated graphene was reported to be 2.4–1.0 i at 532 nm when measured by picometrology based on the work done in [43]. The optical parameter of k can be related to absorption coefficient through Equation (2), where α is the absorption coefficient in cm^−1^ and λ is the wavelength of incident light.

(2) $$\propto =\frac{4\pi k}{k}$$

In the context of a device, the optical responsivity of a graphene-based photodetector is usually poor, which was reported to be in the range of $1\times 10^{-2}\;A/W$ due to limited light absorption at the atomically thin layer [25,33]. These limitations of graphene are being tackled by combining graphene with other materials, especially with 2D materials that form heterostructures, which is possible based on previous studies [6,33], including quantum dots/graphene, MoS_2_/graphene and WSe_2_/graphene heterojunctions in graphene-based photodetectors [25]. Moreover, chemical oxidation of graphene produces graphene oxide (GO), which shows the ability to absorb more light compared to a single layer of graphene at a micrometer thickness. The reduced graphene oxide (rGO) showed tunable carrier transport between electrons and holes dominant transport, in addition to tuning optical properties and bandgap for photodetection application [44]. Nitrogen-doped rGO (N-rGO), which was used to fabricate a photodetector with a metal–semiconductor–metal configuration had a reported photoresponsivity of 0.68 A/W at 1 V that was two orders of magnitude higher than pristine graphene [38], which also indicated the potential self-powering application due to excellent performance at low operating bias.

### 3.2. Molybdenum Disulfide (MoS_2_)

The family of TMDs gained most attention among 2D materials [29]. Generally, TMDs have hexagonal properties that absorb light at the visible to infrared spectrum [25]. They are promising semiconducting materials with ultra-thin bodies that enhance electrostatic gate control and carrier confinement with tunable bandgaps as well as diverse band-alignment with lack of surface dangling bonds [29]. This review emphasizes one of the famous and widely studied TMD materials, which is molybdenum disulfide (MoS_2_), which has Mo atoms sandwiched between two layers of S atoms and has natural availability with ambient/environmental stability [23,29].

MoS_2_ with a thickness of 0.65 nm at the monolayer [29] preserves more than 80% of it transparency, which is considered suitable for wearable applications by industry [18]. It appears as n-type material in nature [33] with the ability of light absorption at the range of visible to near-infrared [25], a large work function of 5.1 eV [45] as well as mechanically stability and transparency for flexible and transparent electronic systems [29]. The most attractive property of MoS_2_ is the adjustable bandgap from 1.2 eV to 1.8 eV when the material changes from the bulk condition to a monolayer, where it transforms from indirect to direct bandgap material, respectively, due to the quantum confinement effect. Therefore, MoS_2_ has thickness-dependable optical properties [11,30,46]. The bandgap changes from a single MoS_2_ monolayer to five layers and remains constant from five layer onwards, where it behaves as a bulk material [29,33]. Thus, it can be highlighted that the bandgap of MoS_2_ increases as the material thickness decreases. The electron affinity of MoS_2_ is also dependent on the number of layers. The electron affinity values that were reported for monolayer MoS_2_ were 4.0 eV [47], those of three layers of MoS_2_ were 4.0 eV [13] and those of multilayers (28 nm) were 4.1 eV [10]. All the values were obtained based on the fabricated devices.

The thickness of the MoS_2_ layer must be carefully chosen for optimum performance, based on application, because the thickness determines the contact Schottky barrier height (SBH), contact resistivity and carrier mobility [29]. SBH reduces from 0.6 eV to 0.3 eV when the thickness of MoS_2_ increases from a monolayer to five layers. Since reduced SBH is preferable for injection of charge carriers, a thicker MoS_2_ will suit this requirement, but a thicker structure is disadvantageous due to intercoupling resistance. Thus, the preferable thicknesses are in the range of 5 nm to 8 nm, which is about seven to twelve layers. The contact resistivity for MoS_2_ with thickness below five layers increased drastically due to quantum confinement-induced structure modification and increased slightly for thickness with five layers and above due to increments in the interlayer resistances. This clearly emphasizes again that the thickness of MoS_2_ must be selected carefully for optimum performance. Moreover, carrier mobility of monolayer MoS_2_ is lower in fewer layers because both the top and bottom surfaces are exposed to the environment that introduces scattering effect. The intrinsic electron mobility is highest in monolayer MoS_2_ because the effective mass of electron is reduced from 0.551 m_o_ to 0.483 m_o_ when MoS_2_ is thinned from bulk to the monolayer. Though the above reviews suggests that thicker MoS_2_ has better properties, it may not be practical for optoelectronic devices because they opt to utilize the direct bandgap nature that comes from the monolayer MoS_2_. Thus, an alternative approach is needed to tackle this issue, and the thickness of MoS_2_ must be tailored accordingly.

MoS_2_ with different thicknesses can tune the light absorption wavelength where a multilayer MoS_2_ phototransistor shows a wide spectrum of detection ranging from ultraviolet to near-infrared, as reported in [48]. As in the previous work by Li et al., the absorption coefficient for a few layers of MoS_2_ was reported to be 0.6 × 10^6^ cm^−1^, and the incident light was mainly absorbed by this layer, which had a thickness of 28 nm because the absorption length was 17 nm [10]. Changes in photoconductivity, absorption spectra and photoluminescence occurred in MoS_2_ with the transformation from direct bandgap to indirect bandgap with variation in the thickness from monolayer to bulk, respectively [49]. The multi-layers of MoS_2_ with thicknesses of 150–165 nm, showed a better rectifying effect compared to MoS_2_ with a thickness ranging from 70 nm to 120 nm in dark condition [50]. Moreover, the large drain current at zero voltage indicated the presence of a photovoltaic effect, which can be attributed to the self-powering effect. The thickness of MoS_2_ is crucial because it influences the optical transparency of the material, which will contribute to the generation rate, as proven in [51,52] for solar cell applications. Optical transparency of MoS_2_ drops from 90% to 40% when the thickness of MoS_2_ is increased from a monolayer to six layers. However, in the context of MoS_2_/h-BN/graphene heterostructures, a multilayer MoS_2_ can establish a vertical depletion region to support tunnelling of photogenerated holes, which is a challenge in monolayer MoS_2_ due to an extremely thin vertical depletion region [10]. Therefore, the thickness of MoS_2_ layers, especially in a vertical device architecture, is a crucial parameter that must be tailored as it trades off the optical properties of the device, which will contribute to the performance of the photodetectors.

### 3.3. Hexagonal Boron Nitride (h-BN)

h-BN is an insulating 2D material that has a lattice constant 1.8% greater than graphene [53]. It is a 2D material that behaves as an insulator with bandgaps varying between 4.5 eV and 6 eV [41], and it has an approximately similar thickness and lattice structure to graphene, which enables both these materials to bind easily [10,11,28,41]. The insertion of h-BN in between graphene and MoS_2_ does not affect the electrical properties of the device at higher V_DS_ (power supply voltage). However, a significant difference is observed when the voltage drops to zero bias. The photocurrent of the MoS_2/_graphene photodetector depresses at zero bias and approaches a similar value as the dark current. In contrast, the MoS_2_/h-BN/graphene photodetector demonstrates better photocurrent at zero bias due to the recovery of the photovoltaic effect. The ultra-thin h-BN layer acts as a barrier to hinder the interlayer transport of electrons, while photogenerated holes in MoS_2_ can tunnel through the ultra-thin h-BN. This recovers the internal electric field of the device [10].

The h-BN incorporation with semiconductor materials, especially with 2D materials, is being studied. The main focus of those research works pertains to studying the influence of h-BN materials on the entire device performance and tailoring the thickness of the h-BN layer to obtain optimum photocurrent with reduced dark current, which will later contribute to the photoresponsivity of the devices. This was proven based on the work in [28], where the thicknesses of h-BN flakes were in the range of 3–6 nm, and h-BN, with a thickness of 4 nm, was chosen to ensure an appropriate tunnelling barrier. This is because thicker h-BN layers lower the tunnelling under moderate biasing, whereas thinner h-BN layers contribute to larger leakage. A similar finding was proven in [36], where 5 layers of h-BN showed better power conversion efficiency (PCE) of 7.1% compared to 3 and 7 layers with PCE of 6.92% and 6.38%, respectively, in a graphene/h-BN/GaAs solar cell. Therefore, there is still plenty of room for research investigation regarding the thickness tailoring of h-BN, especially when interacting with different types of materials at both sides depending on the structure of insertion, which has contribution to improving the built-in potential of self-powering photodetection.

### 3.4. MoS_2_/h-BN/Graphene Heterostructures

Heterostructure devices based on vdW interactions combine the benefits from each material, and the internal built-in potential in the heterostructure can induce effective photogenerated carrier separation [25]. Therefore, a comprehensive summary regarding device physics and working principles, device fabrication and previous works involving graphene, MoS_2_ and h-BN heterostructures is covered in this section in order to understand the advances of these heterostructures in photodetection as well as to identify the research gap for further studies, especially in meeting the requirements for self-powering applications.

#### 3.4.1. Device Physics and Working Principles

**Figure 5:**
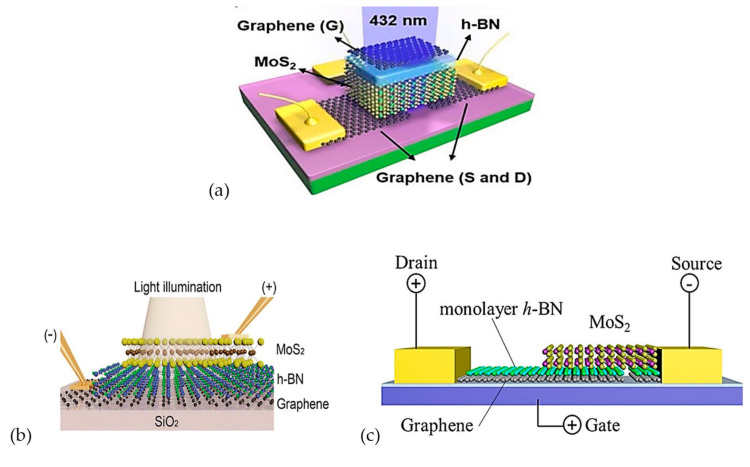
Photodetector architecture with lateral heterostructure of (**a**) graphene/MoS_2_/graphene (Reprinted with permission from [19], American Chemical Society, 2020) and vertical heterostructure of (**b**) monolayer MoS_2_/h-BN/graphene (Reprinted with permission from [54], American Chemical Society, 2016), and (**c**) multilayer MoS_2_/h-BN/graphene (Reprinted from [10], with permission from Elsevier, 2019).

Basically, the architecture of photodetectors can be classified into vertical or lateral heterostructures, and this can determine the current flow in the device. Some architectures of photodetectors based on previous studies are as shown in Figure 5. Devices in Figure 5a are based on lateral heterostructures, whereas those in Figure 5b,c are based on vertical heterostructures. The performance of vertical devices depends on the material thickness, while the performance of lateral devices depends on the channel length, which is the gap between source and drain laterally. The channel length in vertical heterostructures can be as small as the thickness of an atom.

In general, the device physical mechanism of photodetectors can be classified into photovoltaic effect, photoconductive effect, photothermoelectric effect and photobolometric effect. The principles of these mechanisms were covered in the review by Wang et al. [14]. Two or more mechanisms can be incorporated in the same device. As mentioned earlier, the photovoltaic effect is commonly applied for self-powering photodetection. The photovoltaic effect utilizes the built-in potential, which is the internal electric field from the junction (homojunction, heterojunction or Schottky junction) of the device for the photogeneration process [14]. The device physics of graphene-based and MoS_2_-based photodetectors will now be reviewed in this section. Functionalized and hybrid graphene-based photodetectors employ photothermoelectric (PTE), photovoltaic (PV) and photogating (PG) mechanisms for photoresponse, which creates the non-equilibrium distribution of photoexcited carriers and their diffusion and drift in potential gradient. This mechanism depends on the geometry and microscopic carrier dynamics [6]. The PTE effect contributes to light sensing ability in pristine graphene and photoactive areas, which are confined to the junction between two different materials [6].

It is important to understand the operation of a hybrid graphene photodetector in order to improve its performance. Light that is shone onto a hybrid graphene-based photodetector is absorbed by the semiconductor if the photon energy is greater than the bandgap of semiconductor materials, thus creating electron–hole pairs, which form an exciton with an intrinsic efficiency. The large electric field or thermal energy influences the creation of free carriers by overcoming the Coulomb force between electrons and holes, which is associated with efficiency. Lastly, the carriers are transferred between semiconductor and graphene in the presence of a potential barrier at the semiconductor–graphene interface or from a charge trapping mechanism in the semiconductor, which contributes to the charge transfer efficiency [6]. Therefore, the quantum efficiency in the hybrid graphene photodetector depends on the efficiency of intrinsic electron–hole pair generation, the efficiency of creating free charges and the efficiency of transferring carriers between the semiconductor and graphene layers [6]. In this case, the semiconductor behaves as an optical absorber and graphene as the charge transport layer.

The performances of photodetectors with graphene/MoS_2_ heterostructure were studied previously [13,19]. Electrical and optical properties of the heterostructure with a single layer graphene and tri-layer MoS_2_ were studied by Rathi and team [13], where they emphasized the asymmetrical contact effect, because MoS_2_ in this work had contact with titanium at one side and contact with graphene on the other side. This resulted in rectifying characteristics, and the behavior of the device could be tunable with external gate voltage, where the Fermi level of the graphene was shifted upward and downward from the Dirac point with positive and negative gate biasing, respectively. The graphene and MoS_2_ in this work were p-type and n-type, respectively, and the barrier height between them was tunable based on the doping of graphene compared to a fixed barrier between metal and MoS_2_. On the one hand, the graphene/MoS_2_/graphene photodetector in [19] was a lateral device architecture, which improved the photocurrent by controlling the channel length at 30 nm. The similarities in both of these works are that they incorporated h-BN in their devices during the optical analysis and showed improved photocurrent at zero V_DS_ biasing.

The electrical and optical properties from graphene/MoS_2_ heterostructure were improved with the insertion of an h-BN layer in between graphene and MoS_2_, creating a metal–insulator–semiconductor structure, which contributed to rectifying tunnelling devices. Modelling [55,56] and experimental [10,30,47,54,57] works related to graphene/h-BN/MoS_2_ heterostructures at material and device levels were reported in previous studies. The lattice mismatch between these three materials is reasonably small, as reported in [55], where the mismatches at MoS_2_/h-BN and h-BN/graphene were 4.5% and 2%, respectively, which was negligible and preserved their intrinsic electronic properties because the layers were combined via weak vdW energy [56]. Moreover, formation energy in pristine MoS_2_ is higher than doped MoS_2_ at values of −2.3 eV and −1.87 eV at an electric field of 0.1 V/Å, respectively, which also indicates a stable adsorption structure sensitive to the direction of the electric field [55]. This work showed that the external electric field shifted the Dirac cone of graphene from 0 eV to 0.37 eV above the Fermi level, indicating that the graphene was p-doped as a result of field-tunable carrier density.

**Figure 6:**
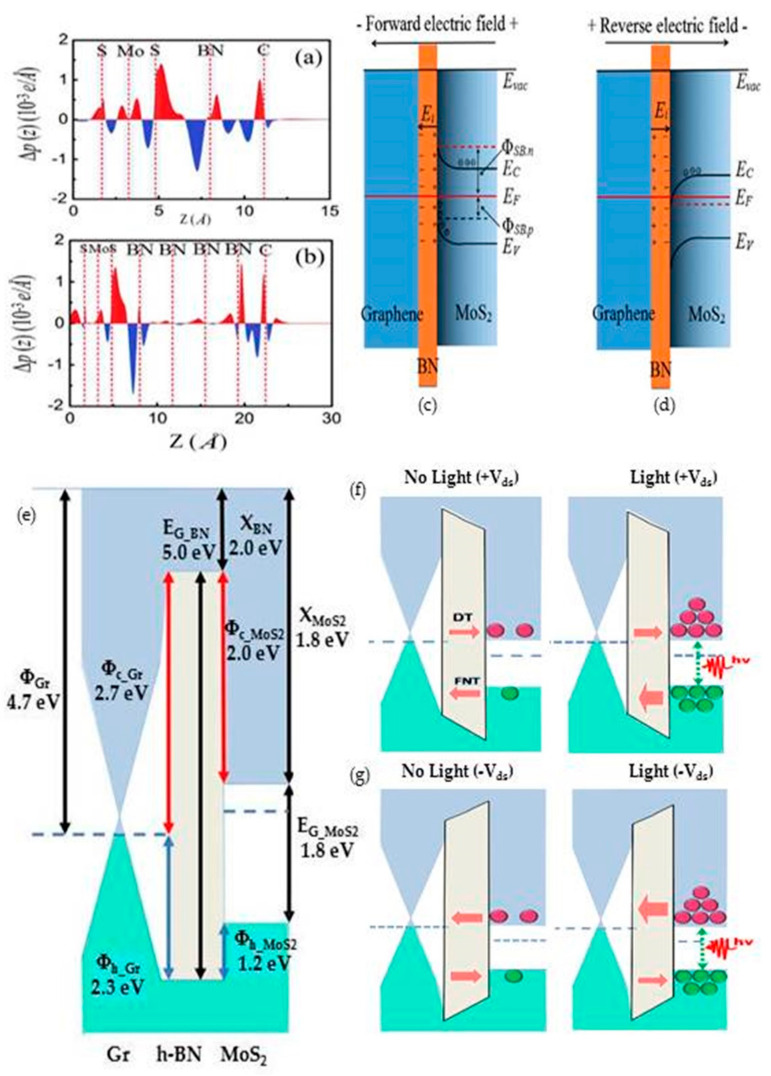
Electron density difference along the z-direction perpendicular to the interface of graphene/h-BN/MoS_2_ heterostructure with (**a**) one layer and (**b**) four layers of h-BN, respectively. The red and blue colors indicate electron accumulation and depletion, respectively (Reprinted from [56], with permission from Elsevier, 2018). Band diagram of graphene/h-BN/MoS_2_ heterostructure under (**c**) forward bias and (**d**) reverse bias (Reprinted from [56], with permission from Elsevier, 2018). Band diagram graphene/h-BN/MoS_2_ photodetector at (**e**) flat band model as well as biasing of (**f**) positive drain voltage and (**g**) negative drain voltage by considering both with and without light (Reprinted with permission from [47], American Chemical Society, 2017).

Moreover, the degree of charge transfer was highest in graphene and lowest in h-BN, which indicated that h-BN is the barrier for charge transfer between graphene and MoS_2_ [55]. Thus, the thickness of h-BN is influential to the amount of charge transfer between graphene and MoS_2,_ which was proven through the work done by Liu in [56] that contributes to device performance, especially in terms of current flow [57]. The work functions of graphene and MoS_2_ were 4.2 eV and 5.8 eV, respectively, which depicted that the Dirac cone was shifted 0.3 eV above the Fermi level, and it was an n-type graphene layer. This resulted in the accumulation of some charges on the right side of h-BN, and some were depleted in the left side. The key finding in terms of dimension was that the intensity of charge transfer in the inner h-BN interlayer was weakened due to the formation of large contact resistance, as shown in Figure 6, when the thickness of h-BN was increased from one layer to four layers.

In order to better understand the electrical operation of devices with graphene/h-BN/MoS_2_ heterostructures, it is important to review the band diagrams of this heterostructure at negative and positive biasing. A potential step was created between graphene and MoS_2_ due to the difference in work function in both the materials. When both graphene and MoS_2_ were brought together, a spontaneous flow of electrons from graphene to MoS_2_ occurred due to conduction band bending of MoS_2_ at the interface. However, the h-BN insertion blocked part of the electron movement that resulted in a built-in electric field because of the formation of a dipole at the interface of the h-BN layer. The changes in the band diagram with applied external electric field are shown in Figure 6c,d [56]. The positive charges accumulated at the h-BN and MoS_2_ interface during forward biasing caused an upward bending of the conduction band and valence band, which resulted in the formation of a barrier for electron flow from graphene to MoS_2_, and this is expected to contribute to good rectifying characteristics. On the other hand, reverse biasing led to the accumulation of negative charges at the h-BN and MoS_2_ interface, which resulted in downward band bending of the conduction band of MoS_2_ that caused an ohmic contact.

Moreover, the band diagram of graphene/h-BN/MoS_2_ heterostructure was also analyzed in the context of a photodetector, as shown in Figure 6e,f [47]. The energy diagram in Figure 6e is a MoS_2_/h-BN/graphene heterostructure at a flat band model with material parameters of electron affinity, bandgap, electron tunnelling barrier, hole tunnelling barrier and work function of graphene. Based on this figure, it can be interpreted that the graphene in the model is a p-type material, as the Dirac point was shifted 0.2 eV below the Fermi level, which results in a work function of 4.7 eV. The hole tunnelling barrier was 1.2 eV, whereas the electron tunnelling barrier was 2.7 eV. The direct tunnelling of electron from graphene layer was blocked by the high h-BN barrier of 2.7 eV, and the Fowler–Nordheim tunnelling of the hole was negligible due to insufficient minority carriers in MoS_2_ during the positive biasing without light conditions that resulted in low dark current. However, photogenerated carriers in MoS_2_ from the photon absorption during the positive biasing with the light condition increase the hole population in the valence band of MoS_2_, which allows the hole tunnelling from MoS_2_ to graphene that is attributed to the increment of illuminated current. A similar pattern was observed in negative drain biasing except for some photogenerated electrons that tunneled through the high electron barrier height between graphene and MoS_2_. Based on these band diagrams, it can be concluded that h-BN layer acted as a barrier for electrons that suppressed the dark current and allowed hole tunnelling during illumination that contributed to a high I_on_/I_off_ ratio, which was also supported by the work done in [36]. It was claimed that the enhancement in performance of light-detecting device with h-BN insertion could be seen based on the high on/off ratio, which was obtained due to the low dark current. The dark current decreased with the increment of h-BN layers but with a trade-off in the photocurrent. Therefore, it is important to tailor the thickness of h-BN to obtain optimum on/off ratios [36].

#### 3.4.2. Device Fabrication

Material synthesis and device fabrication, especially involving graphene, MoS_2_ and h-BN, were reviewed extensively in [6,26,27]. Graphene can be obtained via micro-mechanical cleavage of bulk graphite, CVD and chemical functionalism, as reviewed in [6]. High quality material composition can be effectively fabricated with a highly controlled process via the CVD method, which is well established. The authors also reviewed non-destructive techniques in forming hybrids and heterostructures between graphene and other materials for photodetectors, which are spin-coatings of nanoparticles or quantum dots and stacking of different 2D materials in vdW assemblies to preserve high carrier mobility and enhance light absorption in photodetectors [6]. The vdW forces between layers in bulk 2D materials enable the possibility of obtaining monolayers of these materials via a top-down method such as micromechanical exfoliation and liquid exfoliation. Optical and electrical properties of transferred heterojunctions are usually less superior than the CVD approach due to polymer contamination and deterioration of interfacial quality during the transfer process [27]. In terms of device fabrication, this 2D material can be transferred onto bulk silicon substrates via a simple fabrication approach as follows: spin coating MoS_2_ and Si/SiO_2_ substrates with polymethyl methacrylate (PMMA); removing the sacrificial SiO_2_ sacrificial layer with diluted HF aqueous solution, leaving behind floating PMMA/MoS_2_; transferring PMMA/MoS_2_ onto a new substrate; and finally removing PMMA with acetone while having MoS_2_ film on the new substrate [9].

**Figure 7:**
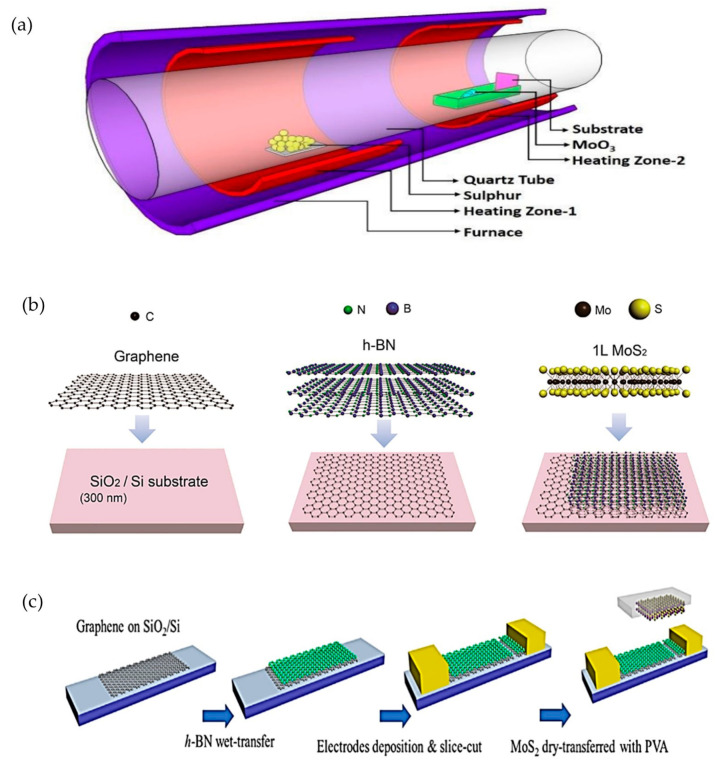
(**a**) Synthesis of vertically aligned MoS_2_ on graphene/SiO_2_ (Republished with permission of Royal Society of Chemistry, from [59]; permission conveyed through Copyright Clearance Center, Inc., 2019). Device fabrication of graphene/h-BN/MoS_2_ heterostructure for (**b**) MIS diode (Reprinted with permission from [54], published by American Chemical Society, 2016) and (**c**) self-powered photodetector (Reprinted from [10], with permission from Elsevier, 2019).

A heterostructure of a combination of MoS_2_ and h-BN was studied previously, as reported in [58]. The chemical vapor deposition (CVD) growth of MoS_2_ on top of h-BN was reported to provide smaller lattice strain, low doping level and a clean and sharp interface by retaining the high carrier mobility in MoS_2_. The fabrication of vertical structure graphene/MoS_2_ by Gnanasekar et al. was reviewed in [59]. Graphene that was fabricated by atmospheric pressure CVD of acetylene on Cu foil was later transferred on silicon dioxide wafer by using poly (methyl methacrylate) (PMMA) film. The Cu foil was chemically etched, and the graphene/PMMA was scooped and placed on the wafer. Then, the PMMA film was dissolved using acetone. The MoS_2_ structure was grown onto graphene by using a two-zone CVD reactor, as shown in Figure 7a. Besides directly growing the MoS_2_ layer onto graphene, a mechanical transfer approach can also be used in fabricating a graphene/MoS_2_ structure.

Some device fabrication approaches for MoS_2_/h-BN/graphene heterostructures, as shown in Figure 7, will be discussed in this section. The device in Figure 7b is a novel MIS diode, which is comprised of graphene, 20nm thick h-BN and a monolayer of MoS_2_ that was used to study both electrical and optical properties of the device [54]. The CVD-grown graphene was transferred to the SiO_2_/Si substrate, which was in 2 cm × 2 cm. Then, 20 nm thick CVD h-BN with a size of 1 cm × 1 cm was transferred on the graphene layer, which was followed by transferring CVD-grown monolayer of MoS_2_, with a size of 500 µm × 500 µm, onto the h-BN/graphene sample. The thickness of the entire MIS structure was reported to be less than 30 nm. The top layer was fabricated smaller compared to the bottom layers to prevent current leakage between layers.

On the other hand, Figure 7c shows another approach in fabricating a MoS_2_/h-BN/graphene photodetector by Li et al. [10]. In this device, a commercial CVD-grown graphene on copper with spin-coated poly (methyl methacrylate) (PMMA) was transferred to SiO_2_/Si substrate. Then, monolayer CVD-grown h-BN was transferred on the graphene using the PMMA method. The photolithography technique was used to pattern and deposit the Au/Cr electrode. A non-conductive gap was created between the electrodes by cutting the h-BN/graphene sample with a probe. Lastly, a few layers of MoS_2_, which were mechanically exfoliated, were transferred on the h-BN/graphene sample using the dry transfer method with polyvinyl alcohol (PVA). The thickness of MoS_2_ and h-BN in this device were 28 nm and 0.47 nm, respectively.

The fabrication processes especially related to the 2D materials were not deeply focused on in this section, because intense reviews were done previously. However, it is important to consider and evaluate the material synthesis and device fabrication approach that are being used because they determine the quality which will be translated in the performance of the device. Therefore, all the synthesis and fabrication approaches for the graphene/h-BN/MoS_2_ photodetectors that are being studied in this review paper are highlighted in Table 2. In summary of device fabrication, most of the graphene, h-BN and MoS_2_ were grown using the CVD method and were transferred using the wet transfer method, which is suitable for CVD-grown samples [60]. h-BN and MoS_2_ in some photodetectors were mechanically exfoliated and integrated by using the dry transfer method.

#### 3.4.3. Material Characterizations and Device Performances

**Figure 8:**
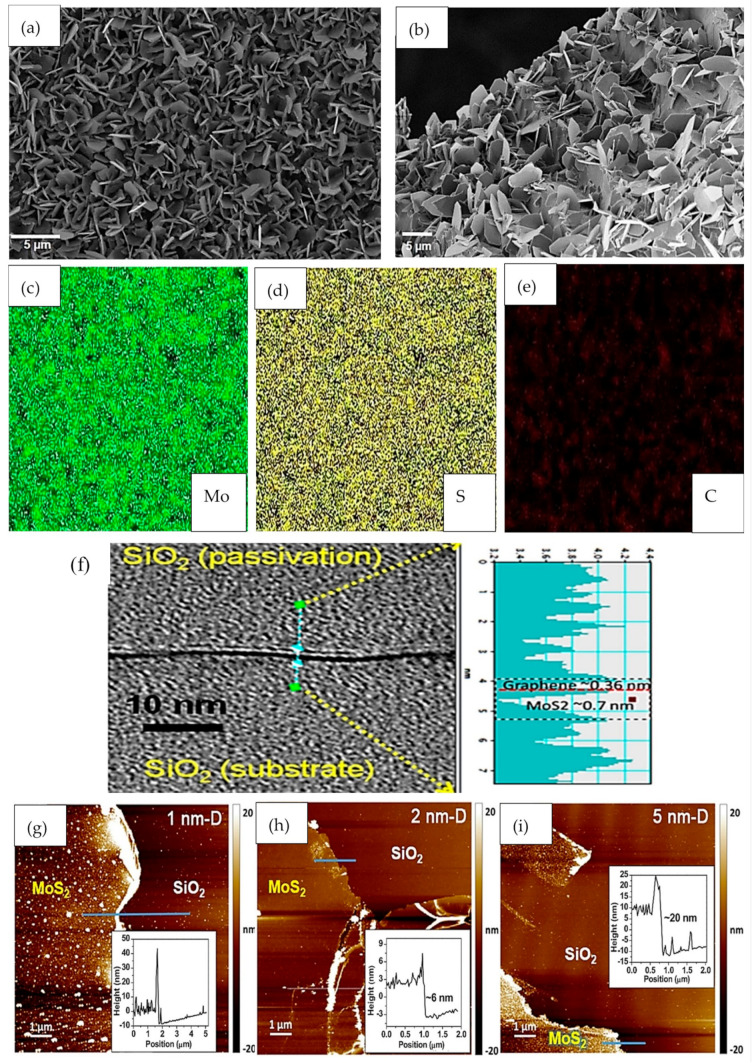
(**a**,**b**) Vertically grown MoS_2_ nanosheet on graphene, (**c**–**e**) EDS elementary mapping of vertical MoS_2_ (element of mapping is mentioned in the bottom right corner) (Republished with permission of Royal Society of Chemistry, from [59]; permission conveyed through Copyright Clearance Center, Inc., 2019), (**f**) high resolution TEM (HRTEM) image of graphene/heterostructures with thicknesses extracted from the intensity profile (Reproduced with permission from [32], Published by Springer Nature, 2014) and atomic force microscope (AFM) images of MoS_2_ films on SiO_2_/Si substrate with pre-deposited Mo thicknesses of (**g**) 1 nm, (**h**) 2 nm and (**i**) 5 nm (Reprinted from [9], with permission from Elsevier, 2018).

The morphology, compositional structures and properties of the MoS_2_, h-BN and graphene heterostructures were analyzed based on optical images, Raman spectroscopy, field emission scanning electron microscope (FESEM), X-ray diffraction (XRD), X-ray photoelectron spectroscopy (XPS), atomic force microscope (AFM), transmission electron microscopy (TEM) and scanning electron microscopy (SEM), as reported in the previous works. FESEM analysis was used to study the morphology of a vertically-aligned CVD-grown MoS_2_ nanosheet on graphene, as reported by Gnanasekar et al. [59], as shown in Figure 8a–e. Vertical MoS_2_ nanosheets with thickness and length of 56 nm and 5 µm, respectively, were reported, and the EDS elementary mapping confirmed homogenous coverage of MoS_2_ on the graphene layer. Moreover, the high resolution TEM (HRTEM) approach can also be used to study the cross-section of a MoS_2_/graphene device, such as shown in Figure 8f. This device was capped with SiO_2_ as a passivation layer, and the intensity profile shows the thicknesses of graphene and MoS_2_ at values of 0.36 nm and 0.7 nm, respectively, which indicated that both the materials in the heterostructure were monolayers.

**Figure 9:**
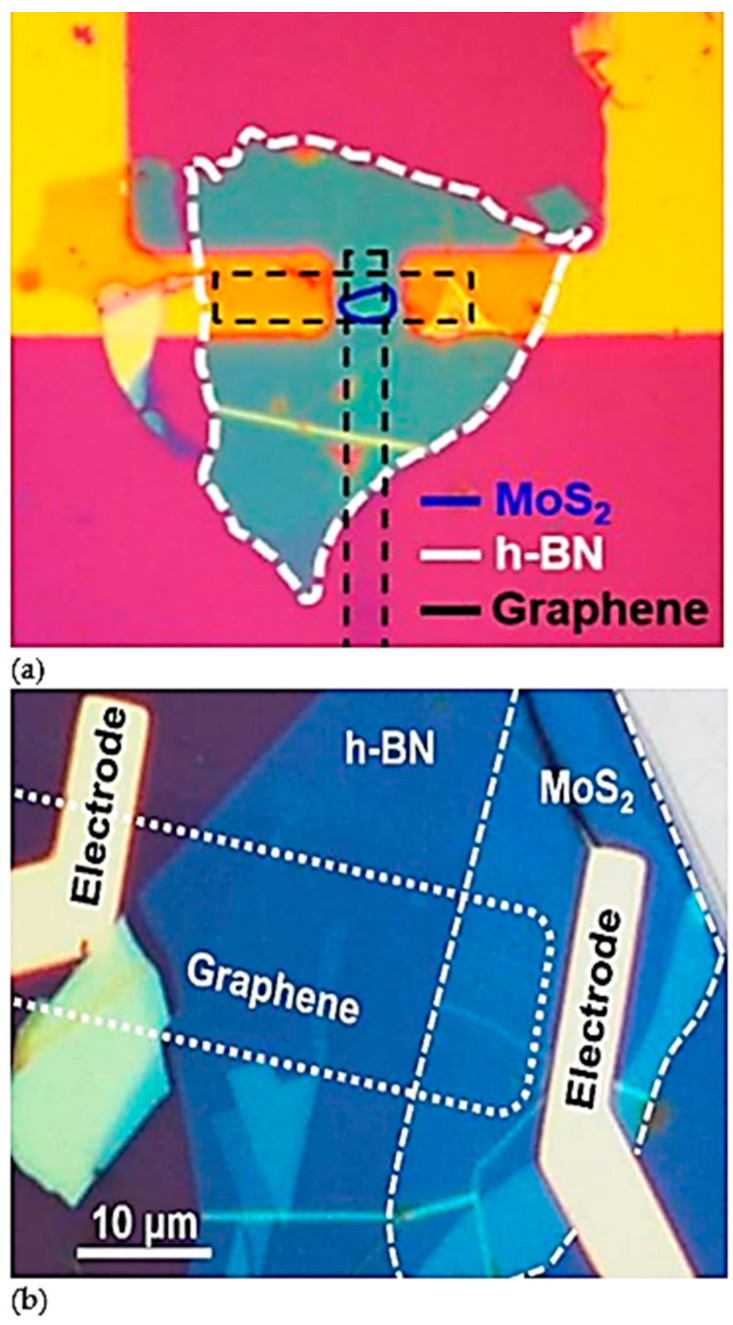
Optical images of MoS_2_/h-BN/graphene heterostructures. (**a**) Lateral graphene/MoS_2_/graphene device with channel length of 30 nm. h-BN serves as the dielectric layer for transparent graphene gate electrode (Reprinted with permission from [19], American Chemical Society, 2020). and (**b**) The dotted line is graphene layer at the bottom, the dashed line is monolayer of MoS_2_ at the top and 11 nm thick h-BN layer between them (Reprinted with permission from [47], American Chemical Society, 2017).

Moreover, MoS_2_ films with various pre-deposited Mo thicknesses of 1 nm, 2 nm and 5 nm that were employed for flexible photodetector in [9] were characterized based on AFM techniques. Images from the AFM approach were used to determine the quality and thicknesses of MoS_2_ films that were transferred, as shown in Figure 8g–i. The flat and continuous surface from samples of 2 nm and 5 nm, as shown in Figure 8h–i, deduced that the thicknesses of MoS_2_ films were 6 nm and 20 nm, which are about 9 and 31 layers, respectively. The large roughness and defects in the sample with pre-deposited Mo with thickness of 1 nm showed that the sample was of a bad quality, and the thickness of MoS_2_ film was difficult to be determined. Aside from that, the presence and formation of MoS_2_, h-BN and graphene heterostructures can also be confirmed through optical images such as shown in Figure 9. The lateral device architecture that is shown in Figure 9a is comprised of a graphene/MoS_2_/graphene heterostructure with a channel length of 30 nm, and h-BN served as the gate barrier in this device. However, devices in Figure 9b show the MoS_2_/h-BN/graphene heterostructures with vertical device architecture, where the dotted and dashed lines indicate the presence of graphene and MoS_2_ layers, respectively.

**Figure 10:**
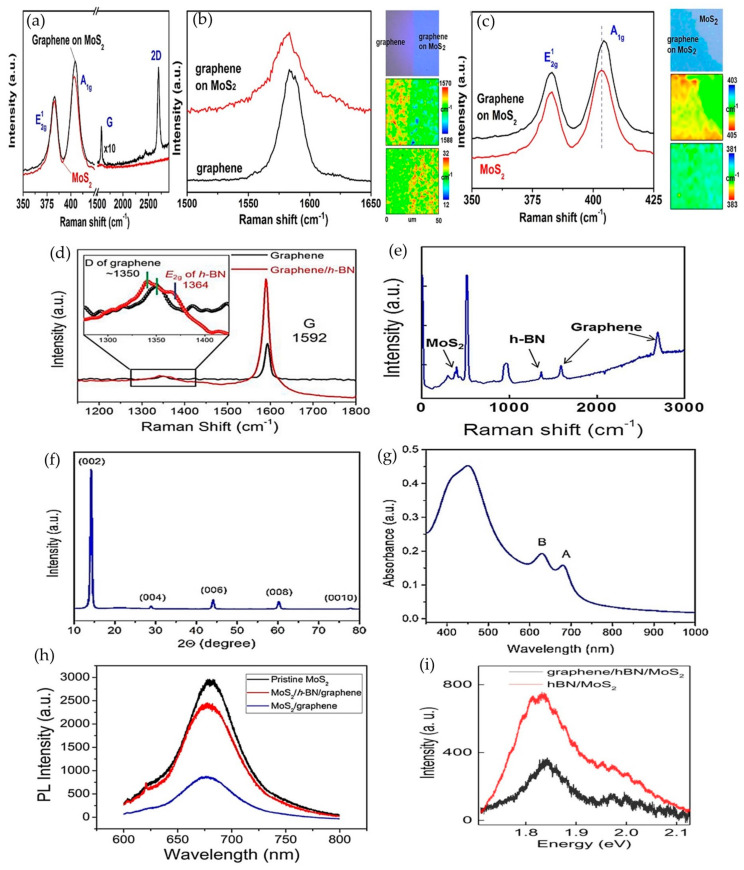
Raman spectra of (**a**) graphene/MoS_2_ heterostructure with comparison to (**b**) monolayer graphene, (**c**) monolayer MoS_2_ (Reproduced with permission from [32], Published by Springer Nature, 2014), (**d**) h-BN/graphene (Reprinted from [10], with permission from Elsevier, 2019) and (**e**) MoS_2_/h-BN/graphene light (Reprinted with permission from [47], American Chemical Society, 2017). Characterization of V–MoS_2_ nanosheets with (**f**) XRD diffraction pattern and (**g**) UV–Vis absorption spectrum (Reprinted from [61], with permission from Elsevier, 2019). Photoluminescence spectrum of (**h**) pristine MoS_2_, MoS_2_/h-BN/graphene and MoS_2_/graphene heterostructure to denote the dynamics of photogenerated electrons (Reprinted from [10], with permission from Elsevier, 2019) and (**i**) graphene/h-BN/MoS_2_ and h-BN/MoS_2_ for optoelectronic applications (Republished with permission of IOP Publishing, Ltd., from [30]; permission conveyed through Copyright Clearance Center, Inc., 2020).

On the other hand, the properties of the materials can be determined based on the intensity peaks in Raman spectroscopy. A monolayer graphene has two Raman peaks, which are G (1580 cm^−1^) and 2D (2960 cm^−1^), and shows in-plane and out-plane vibration. The number of graphene layers can be determined based on the ratio of intensities of 2D and G peaks, position and shape of the peaks. The G peak can indicate the number of graphene layers, and the frequency of this peak decreases as the number of layers increases [41]. The D peak in graphene indicates the disorder, and the level of disorder is indicated based on the ratio of intensities of D and G peaks. In contrast, MoS_2_ has two Raman peaks, which are E^1^_2G_ and A_1G_, and the difference between these two peaks indicates the number of layers. The Raman peak for h-BN crystal is 1336 cm^−1^ and experiences blue and red shifts due to shorter phonon modes in single layer h-BN, and random strain due to stripping process in double h-BN, respectively [41]. Raman analysis for MoS_2_, h-BN and graphene heterostructures based on previous works are shown in Figure 10. The intensity of Raman spectra in Figure 10a was increased by 10 for more visibility. The MoS_2_ in this heterostructure was confirmed to be a monolayer based on the difference between Raman E^1^_2G_ and A_1G_ peaks, which is about 19 cm^−1^. The G and 2D peaks for graphene were reported to be 1581.5 cm^−1^ and 2695.9 cm^−1^, which are approximately reaching the values of monolayer graphene.

The Raman spectra was further analyzed by comparing the findings with respect to monolayer graphene and MoS_2_, as shown in Figure 10b,c, to understand the carrier movements in the heterostructure. The broadening of the G peak from 1585.4 cm^−1^ in graphene to 1581.7 cm^−1^ in the graphene/MoS_2_ heterostructure depicted a red-shift, which resulted from increases of the Fermi level with Raman laser exposure, where the photogenerated electrons were injected to the graphene layer. A similar approach was used to study photogenerated carrier movements in the MoS_2_ layer. The Raman spectra in Figure 10c shows the 1 cm^−1^ up-shift in the A_1G_ peak with a narrower width in the graphene/MoS_2_ heterostructure compared to MoS_2_, which indicated less n-doping in the MoS_2_ layer. The changes in Raman spectra led to the conclusion that the photogenerated electrons move from the MoS_2_ layer to the graphene layer in the graphene/MoS_2_ heterostructure, whereas photogenerated holes were trapped in the MoS_2_ layer. The Raman spectra analyses for graphene/h-BN and MoS_2_ heterostructures were covered as well in this section. Figure 10d shows the Raman spectrum of graphene and graphene/h-BN based on the self-powered photodetector that was reported in [10]. The D and G peaks of monolayer graphene were 1350 and 1592 cm^−1^, respectively, and these peaks experienced minor shifts when covered with h-BN, denoting the good contact between h-BN layers though graphene was introduced with lateral tension force from h-BN coverage. Similarly, the Raman spectra in Figure 10e show the presence of all three layers in graphene/h-BN/MoS_2_ heterostructures in the photodetector that were reported in [47].

In addition, the crystallinity and quality of materials were also confirmed based on XRD, the UV–visible absorption spectrum and the photoluminescence (PL) spectrum. Figure 10f,g shows the XRD and UV–vis spectrum for the vertical MoS_2_ (V–MoS_2_) nanosheet studied in [61]. XRD results were analyzed based on the location of the diffraction peaks (*x*-axis) and diffraction plane (*y*-axis). The most intense peak indicates a strong tendency of adaption to the crystal orientation during the growth process. As in Figure 10f, the diffraction peaks at 14.2°, 28.94°, 44.1°, 60.19° and 77.8° corresponded to (002), (004), (006), (008) and (0010) planes of MoS_2_, which denoted that the V–MoS2 nanosheets were highly (002) orientated. The absorption peaks of A and B were located at 600–700 nm, agreeing with 2H phase V–MoS_2_ nanosheets. Moreover, the photoluminescence (PL) spectra for graphene/h-BN/MoS_2_ heterostructures were studied in previous works [10,30] for characterizing the dynamics of photogenerated electrons. A strong PL peak of monolayer MoS_2_, as shown in Figure 10h, was suppressed when transferred onto the graphene layer, depicting that the recombination process occurs at the graphene layer. The reduced photovoltaic effect in this heterostructure was recovered with h-BN insertion, where the PL peak of MoS_2_/h-BN/graphene was improved compared to the MoS2/graphene heterostructure. This supported the proposal by Li et al. for improving the photovoltaic effect in the MoS_2_/graphene photodetector with h-BN insertion for self-powering applications [10]. Similar findings were reported in Figure 10i and highlighted that the quenching in excitonic peak in the PL spectra resulted from the interlayer charge transfer, especially between graphene and MoS_2_ [30].

The continuation from material characterizations is the analysis of device performances, because these two aspects are interrelated. The device performances of graphene-based photodetectors especially with the heterostructures were reviewed intensively by Shin et al. [15]. They covered the performances of various graphene-based photodetectors including graphene/2D material heterostructures. Their review concluded that the number of graphene layers, chemical doping, photoinduced doping and photogating doping can tune the work function, surface resistance and optical transparency of graphene. They also highlighted the capability of plasmonic technology in enhancing the light absorption in photodetectors and their area of interest focused on photodetectors with graphene as transparent conductive electrodes (TCE). Therefore, this review paper is extended specifically to reviewing the photodetectors/solar cells based on graphene/MoS_2_ and graphene/h-BN/MoS_2_ heterostructures to study their relevance for self-powering photodetections. The MoS_2_/bulk silicon heterojunction photodetector, which was studied by Deng et al., supported and showed that the device fabrication demonstrated good compatibility with the silicon CMOS process while achieving excellent photoresponsivity at 1.7 × 10^4^ A/W with rise and fall times of 1.44 ms and 1.45 ms, respectively, at the near infrared spectrum [62]. This shows that the 2D material has the potential to broaden the detection spectrum up to near-infrared light.

Moreover, a MoS_2_/graphene photodetector that was fabricated in 2015 showed maximum responsivity of 2.06 × 10^3^ AW^−1^ at incident light with an optical power of 14 mW cm^−2^ at a wavelength of 520 nm, which was claimed to be higher than previous work [13]. To justify the benefits of hybridization between 2D materials, a graphene/MoS_2_/graphene photodetector was fabricated and studied by Liu et al. without complex transfer and alignment processes. This lateral device showed a maximum specific detectivity of 10^13^, responsivity of 2 × 10^3^ mA/W and on/off ratio up to 10^6^ (which indicates low dark current) at the visible light spectrum [63]. A similar lateral device approach was used by Lee et al. in 2020 to improve photodetector performance by using short channel and tunable Schottky barrier with a design of graphene/h-BN/MoS_2_/graphene that had a channel length of 30 nm. Light and gate biasing were used to tune the Schottky barrier, which resulted in improved photocurrent and low dark current with a responsivity of 2.2 × 10^5^ A/W and a detectivity of 3.5 × 10^13^ Jones [19]. They used the scaling approach by improving the gain of the photodetector by reducing the transit time and decreasing the recombination during carrier movement through a shorter channel length. The correlation between channel length, responsivity, gain and detectivity are expressed in Equation (3), where *L* is the channel length, *µ* is carrier mobility and *V_DS_* is the drain voltage.

(3) $$D^{*}*\propto R\propto Gain=\frac{\tau_{lifetime}}{\tau_{transit}}=\frac{\tau_{lifetime}}{\frac{L^{2}}{\mu V_{DS}}}$$

In 2016, Jeong et al. [54] had studied the photoresponse of a MoS_2_/h-BN/graphene heterostructure as a metal–insulator–semiconductor (MIS) diode when the device was illuminated with visible light at 688 nm with an optical power of 7 mW and a light power density of 2.8 W/m^2^. The thickness of this device structure was a monolayer of MoS_2_ with an h-BN of 20 nm. The entire thickness of this device was below 30 nm. The photocurrent/dark current ratio was around 6.6 with photoresponsivity of 0.3 mA/W as well as decay and rise time of 11 s and 10 s, respectively. The findings were claimed to be ten times higher than the graphene/MoS_2_ heterostructure that was reported earlier.

In the following year, a similar type of study on photodetector with single-layer MoS_2_ and graphene strips that sandwiched h-BN was reported by Vu et al. [47]. The thickness of h-BN was varied and optimized at 7 nm, which contributed to a high photocurrent/dark current ratio of more than 10^5^, a high photoresponsivity of 180 A/W and an ultra-high photo detectivity of $2.6\times 10^{13}$ Jones at a wavelength of 405 nm. Although the two studies could not be compared directly, as the operating conditions, such as optical wavelength and power density, were different, some of the device parameters that contributed towards the performance of the photoresponse can be analyzed. Firstly, is the fabrication process, where all the materials of the MIS diode in [54] were grown with the CVD process, while the latter work in [47] was prepared with CVD grown graphene and mechanically exfoliated h-BN and MoS_2_ that was transferred with the dry transfer approach. As mentioned earlier, mechanically exfoliated materials have better crystal quality with freshly cleaved surfaces [57]. Secondly, a noticeable difference between these two works is the dimension of the layers, which is the focus of interest in this paper. Both the works used monolayer MoS_2_, which has a thickness of 0.65 nm. However, the thicknesses of graphene and h-BN were different. These dimensions are considered as crucial, since the layers in the device are vertically structured.

**Figure 11:**
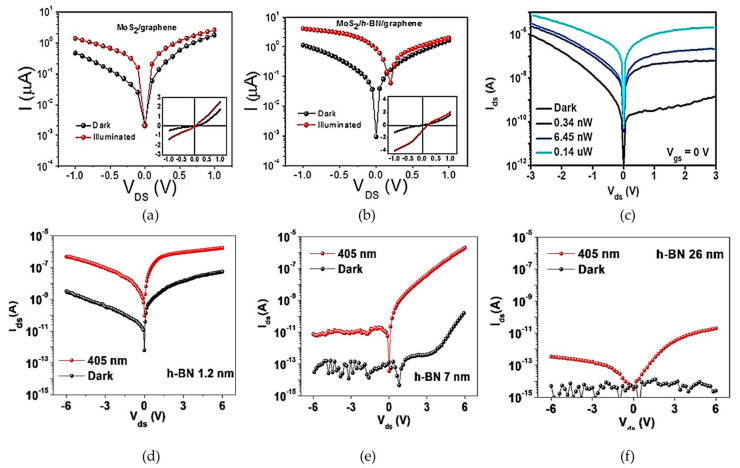
I–V characteristics of MoS_2_/h-BN/graphene photodetectors. Vertical MoS_2_/graphene photodetector (**a**) without and (**b**) with h-BN insertion (Reprinted from [10], with permission from Elsevier, 2019). (**c**) Lateral graphene/MoS_2_/graphene photodetector (Reprinted with permission from [19], American Chemical Society, 2020). MoS_2_/h-BN/graphene photodetector with varied thickness of h-BN at (**d**) 1.2 nm, (**e**) 7 nm and (**f**) 26 nm (Reprinted with permission from [47], American Chemical Society, 2017).

The I–V characteristics of photodetectors with MoS_2_/h-BN/graphene that were discussed previously are compiled in Figure 11. The insertion of h-BN to improve the photovoltaic effect in MoS_2_/graphene photodetector was proposed by Li et al. [10], where they showed three orders of improvement in the photocurrent at zero bias. Previously, Rathi et al. inserted a few layers of h-BN between the tri-layers of MoS_2_ and single layer graphene in their optical studies. However, they claimed that the h-BN had no active role in their device because the single layer graphene was in direct contact with the MoS_2_ layers. Figure 11a,b show the findings from [10], where the influence of h-BN insertion can be clearly seen. The suppression of dark current and photocurrent were at the same value at zero biasing without the h-BN insertion. This is because the barrier height between MoS_2_ and graphene was at 0.23 eV with small bending of 0.09 eV in MoS_2_, which allowed leakage of electrons from MoS_2_ to graphene that resulted in increased dark current, even at zero biasing. Thus, this leakage was stopped with the insertion of h-BN, where it acted as a barrier to block the interlayer movement of electrons and only allowed tunnelling of photogenerated holes from MoS_2_ to graphene with the internal electric field. Therefore, the dark current was suppressed more at zero bias, which resulted in an on/off ratio of 1000. This was considered as recovering the photovoltaic effect in the device, which is beneficial for self-powering photodetection.

Nevertheless, dark current was also lowered in the lateral graphene/MoS_2_/graphene photodetector [19], as shown in Figure 11c, where h-BN acted as a gate dielectric layer in this device. This controlled the performances of this device by ensuring a smaller channel length of 30 nm and modulating the Schottky barrier between graphene and MoS_2_. The influence of h-BN thickness on the performance of photodetectors is shown in Figure 11d–f, where the thickness of h-BN was varied at 1.2 nm, 7 nm and 26 nm. A thin h-BN allows high leakage, which increases the dark current, whereas a thicker h-BN barrier suppresses both the dark and photocurrent. Both these conditions deteriorate the performance of the photodetector. Therefore, it is important to tailor the optimal thickness of the h-BN layer to ensure optimum performances of the photodetector, as shown in Figure 11e. 

A list of photodetectors that utilizes the graphene, MoS_2_ and h-BN heterostructures that were discussed in this section are tabulated in Table 2 with their figures of merit.

### 3.5. Prospect of Materials towards Self-Powering Application

The prospect of 2D materials with their challenges and market study was reported in [65]. Based on the reviews related to 2D material-based photodetectors, the venture into this field was started in 2014 and is being continued until now, especially for the graphene/h-BN/MoS_2_ heterostructures. The ultimate goal of these studies was to improve the performance of the device based on the figure of merit discussed in Section 2 by evaluating the properties of the heterostructure as a system. Since the devices were vertically stacked, the vertical dimension, which is the thickness of each layer, is a crucial parameter. Excellent diode characteristics with a rectification ratio of 7.2 × 10^2^ at ±1 V were demonstrated in thick MoS_2_ compared to linear characteristics in thin MoS_2_, which has ohmic contact between MoS_2_ and the Au electrode. A multilayer MoS_2_ transistor has increased current under illumination of red, green and UV light in vacuum, which is dominant in p-type transfer and n-type conduction under dark and illuminated conditions, respectively [50]. This study highlighted the potential of thick multilayer MoS_2_ for self-powering applications, indicating the importance of tailoring the thickness of the absorbing layer. The optimum individual thickness of each layer was reported with an analysis of their properties and performances. However, tailoring the thicknesses of all the layers to obtain one optimum system is still lacking and needs much attention. Figure 9 shows the I–V characteristics from MoS_2_/h-BN/graphene photodetectors that have potential in contributing to self-powering application.

## 4. Plasmonic Implementation in Photodetector

Modification of the photodetector detection surface with light sensitizer effects such as quantum dots [66,67], dye and noble metal nanoparticles (NPs) [61] demonstrated an improvement in the performance of photodetectors, especially in terms of responsivity [68]. Studies reported that usage of plasmonic metal NPs improves device performance, because strong photon scattering and absorption are induced by increased localized surface plasmon resonance (LSPR) that occurs during photon illumination onto metal NPs [61,69]. LSPR refers to the interaction of electromagnetic waves that are confined on metallic nanostructures during illumination and that are sensitive to the refractive index changes around the metallic structure [70]. It is also applicable for self-powering photoelectronic applications with a broad spectrum of light [71,72,73]. A graphene coating with plasmonic has been used extensively due to its superior optical, mechanical and electrical properties, such as high electron mobility, high surface to volume ratio and stable atomic structure [45]. In this section, the device physics and previous work related to plasmonic incorporation for light detecting application, especially with graphene and MoS_2_ structures, will be reviewed.

### 4.1. Device Physics

The interaction between the electromagnetic wave and free electrons in the metal is known as surface plasmon resonance (SPR), in which the light (electromagnetic wave) excites free electrons in the metal to achieve selected absorption or reflection, producing an enhancement in light intensity and trapping electromagnetic energy in deep subwavelength volume, area or length [74,75,76]. Studies showed that modification of the detection area with nanostructures or nanoparticles contributed to the improved performances in the photodetector. Furthermore, it is possible to load nanoparticles and quantum dots on the surface of 2D materials [4]. Previous studies also highlighted that size [77], type, pitch, orientation array and shape of nanoparticles influenced the energy conversion efficiency and concluded that spherical shape nanoparticles had better optical absorption and near field enhancement, which indicated that those nanoparticles influenced more photon absorption/scattering in the device [77,78]. NPs of some noble metals such as Au, Ag and Pt are inert substances that show the effect of LSPR under illumination for applications like surface-enhanced spectroscopy, biomolecule detection and light transmission. The performances of photodetectors are still not up to expectations, despite the intense studies on LSPR in photodetection, because of difficulties in controlling the ideal parameter of noble metal NPs for best performance [68]. Resonant intensity caused by the effect of LSPR is sensitive to the size and shape of particles as well as dependent on the wavelength of light irradiation. On the other hand, a previous study also showed that the incorporation of Au NPs on hybrid 2D materials, especially in this case where Au NPs were decorated on MoS_2_/graphene devices, showed better sensitivity compared to embedding Au NPs on either MoS_2_ or graphene [79]. Au nanoparticles or periodic nanoarrays on few layers of MoS_2_ showed a threefold enhancement of photocurrent response due to enhanced near-field oscillation and the scattering effect of nanoparticles [80].

**Figure 12:**
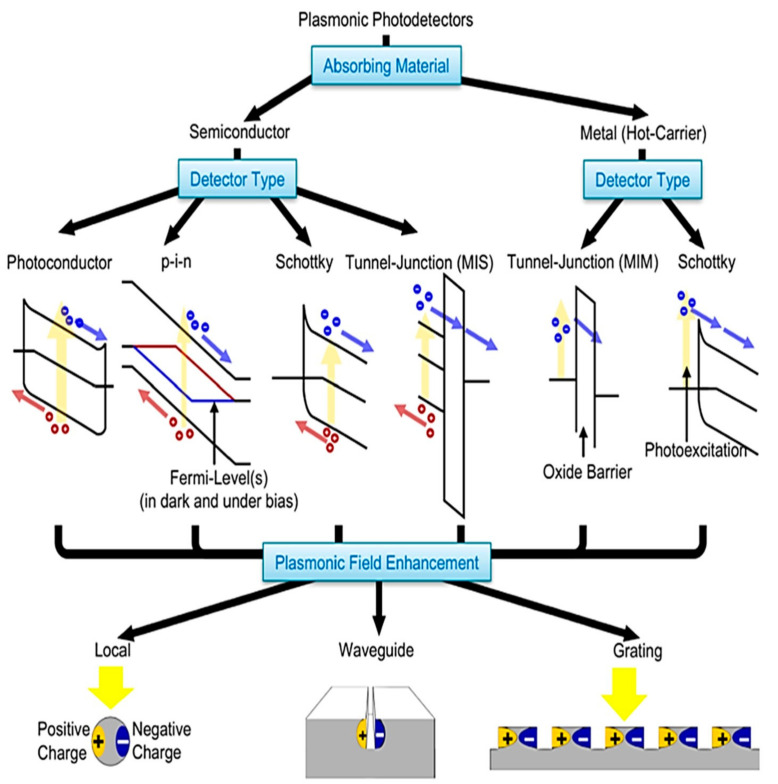
Simplified classification diagram of plasmonic photodetector (© 2018 IEEE. Reprinted, with permission, from [81]).

A previous review of plasmonic photodetectors highlighted that the embedment of plasmonic coupling concept into the photodetector opens up the potential for shrinking the size of photonic devices to the nano scale with a higher speed and wider frequency spectrum of applications by employing the ability of the metal to constrain light at deep sub-wavelengths [81]. The utilization of the plasmonic effect, especially in the photodetection application, was reviewed in depth by Dorodnyy et al. in [81], and the plasmonic-based photodetectors were classified based on Figure 12. It was shown that the absorption material in plasmonic photodetectors rely on the semiconductor structure or metal (for hot-carrier photodetector) that can be further classified based on the operation scheme, which is a photoconductor detector, p–i–n photodetector, tunnel-junction detector and Schottky-detector. The plasmonic effect in these detectors can be enhanced by a localized plasmon polariton in a metallic nanoparticle, a surface plasmonic polariton (SPP) in a waveguide and a grating type plasmon. Monolayer 2D materials in photodetectors, which are atomically thin and could not sufficiently absorb light, needed some kind of mechanism to enhance the photon absorption. Thus, previous work reported that the utilization of localized plasmonic enhanced the performance of the device.

Incorporation of plasmonic nanoparticle scattering towards performance enhancement in photovoltaic and photodetector devices was discussed in [77] by analyzing the improved photosensitivity and photoresponse based on the coupling of optical and dielectric properties of nanoparticles. A periodically structured Au nanodot array enhances the photocurrent in the photodetector compared to the randomly structured nanoparticles that can be applied in photovoltaic devices [82].

Besides the shapes, size and material of nanoparticles, the location of nanoparticle embedment can influence the LSPR effect in the photodetector. Metal nanoparticles can be embedded based on deposition at the top of the detection medium, integrating below the detecting medium or embedding within the detecting medium [83]. Nanoparticles that are embedded into the detecting medium become charged during illumination due to the absence of a contact electrode, and the discharging process via compensating charge injection from the host medium might be slow. The easiest and simplest approach was to deposit the nanoparticles at the top surface where the light penetrates but is restricted with non-uniform shapes and sizes of nanoparticles.

### 4.2. Material Characterizations and Device Performances

**Figure 13:**
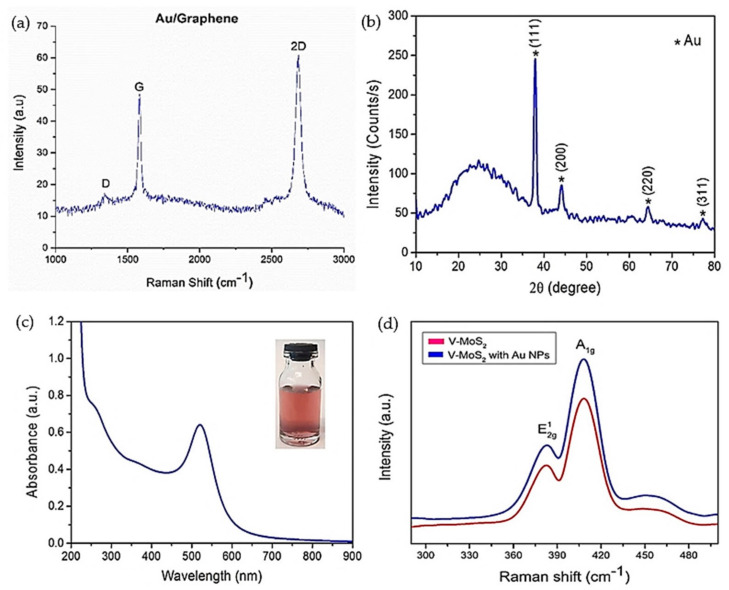
(**a**) Raman spectra of transferred graphene on Au thin film (© [2020] IEEE. Reprinted, with permission, from [84]). (**b**) XRD pattern and (**c**) UV–Vis absorption spectrum of Au NPs as well as (**d**) Raman spectra of V–MoS_2_ with and without Au NPs (Reprinted from [61], with permission from Elsevier, 2019).

Before analyzing the performance of photodetectors with plasmonic coupling, it is important to understand the material characterizations, as these confirm the quality and physics of plasmonic effects. Figure 13a shows the Raman spectra of the transferred graphene layer on the thin Au SPR substrate. Although this device focused on sensing applications, the material characterization can be utilized as fundamental in understanding the plasmonic effect of Au thin film towards the properties of materials, which is graphene in this case. The D, G and 2D of Raman modes in this transferred graphene were, respectively, 1340, 1585 and 2684 cm^−1^ The shift between G and 2D peaks and presence of D peaks in this Raman spectra clearly signify that bilayer graphene, which was obtained based on ratio of peak intensities (I_2D_/I_G_), were transferred on the Au thin film with large defect density [84,85]. On the other hand, the XRD diffraction pattern and UV–Vis spectrum for Au nanoparticles with resulting Raman spectra were investigated in [61] based on Figure 13b–d. The gold nanoparticles, which were synthesized by laser ablation of gold targets, showed XRD diffraction pattern with peaks at 37.74°, 44.09°, 64.34° and 43.20° corresponding to planes of (111), (200), (220) and (311), respectively, with strong adaption to the (111) crystal plane. The UV–Vis absorption spectrum of Au NPs in ethanol medium, as shown in Figure 13c, indicated a single strong surface plasmon resonance at 520 nm. The effect of Au NPs is seen in Figure 13d, where Raman intensity increased, indicating the enhancement of a light-induced electric field near the surface of Au NPs, and this improved the absorption of light in V–MoS_2_ [61].

**Figure 14:**
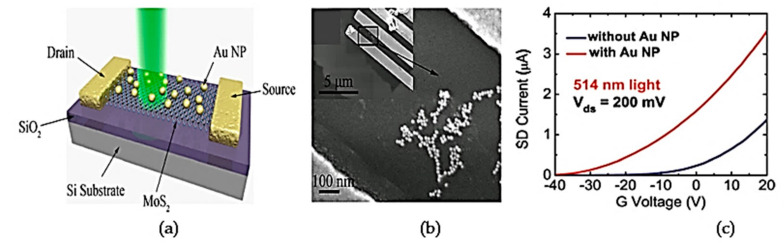
(**a**) Schematic of MoS_2_ transistor with Au NPs decoration, (**b**) scanning electron microscopy image of Au NPs on MoS_2_ and (**c**) the electrical characteristic of MoS_2_ transistor with and without Au NPs (Reprinted from [86], with the permission of AIP Publishing, 2013).

Incorporation of the plasmonic effect with MoS_2_ for device enhancement was reported in 2013 by Lin et al., where the photocurrent in the transistor was improved by decorating the MoS_2_ with Au NPs [86]. Figure 14 shows the schematic view of a MoS_2_ transistor decorated with Au NPs under illumination of light at a wavelength of 514 nm with the scanning electron microscopy image of Au NPs on the MoS_2_ and the electrical characteristic that indicates the improvement of photocurrent with the presence of Au NPs. Interestingly, they highlighted the increment in charge carrier mobility and electron concentration in both dark and illuminated conditions when the device was decorated with Au NPs. This confirmed the potential of utilizing the plasmonic effect in the MoS_2_, which acts as the absorber in the MoS_2_/h-BN/graphene heterostructures.

Work done by Jing et al. in 2017 reported 470% improvement with a responsivity of 2.97 × 10^4^ A/W as well as rise and fall times of 18 s and 7 s, respectively, at an illumination wavelength of 610 nm [68]. They modified the detection surface with Ag NPs and developed the device by using a single layer of MoS_2_ as the channel of the phototransistor. Study of the effect of NP size with light absorption and intensity of the device was also done in this work. Samples coated with Ag NPs at particle sizes from 13 nm to 25 nm showed obvious enhancement of intensity compared to Ag NPs with sizes exceeding 40 nm, which led to decreased intensity due to the shield effect of scattering and metallic absorption. A similar plasmonic approach was used by Gosh et al. in 2019, where Ag NPs at sizes ranging from 5 to 14 nm with a mean size of 11 nm were synthesized and added with organic layers of PEDOT:PSS to fabricate a plasmonic hole-transport-layer enabled self-powered hybrid perovskite photodetector [87]. This work reported a highest responsivity of 0.25 A/W at 400 nm with rise and fall times of 110 ms and 72 ms, respectively. The device showed higher enhanced responsivity in the ultraviolet region compared to the visible region. Responsivity enhancement in the visible region was contributed by improved carrier extraction and transport by Ag NPs, while responsivity improvement in the ultraviolet region was contributed by enhanced optical absorption by Ag NPs. What makes this device special is that it was fabricated entirely at ambient atmosphere with a modified perovskite deposition method, which makes it suitable for practical device fabrication. Both of these works proved the possibility of embedding NPs into 2D materials in order to improve the performance of the device. They also showed that the spectrum of detection of 2D materials with NP plasmonic coupling varied from the ultraviolet to the visible range.

Beside using Ag NPs, a study on a photodetector employing porous Ag/TiO_2_ by [87] was reported in 2019 for the plasmonic hot-electron effect. Such a device is a self-powered photodetector which can operate at zero voltage. The detectable photocurrent in this type of device is caused by incident light that excites surface plasmons on the porous Ag, which later decay to be hot electrons and holes. This work showed responsivity of 3.3 mA/W as well as rise and fall times of 112 µs and 24 µs, respectively. The responsivity was comparable to the previous reported photovoltaic hot-electron photodetectors, and the response speed was better than that previously reported plasmonic hot-electron photodetectors. The device had a broad response spectrum from the visible to near-infrared region.

On the other hand, Reddeppa et al. in 2019, studied Au NP-functionalized rGO/GaN nanorods (NRs) [88]. Spherical shaped Au NPs with a uniform size of 50 nm were used to study the performances of the device in detecting light at ultraviolet (328 nm) and visible (516 nm) regions, as well as for gas detection. They reported photocurrents of 53.92 μA and 411.57 μA at wavelengths of 516 nm and 382 nm, respectively. The findings of this study clearly showed fast current increment and decrement with rise and fall time of 0.43 s and 0.38 s, respectively, at the ultraviolet region compared to rGO/GaN NRs. Au NPs with enhanced photoresponse properties in this device contributed to interband transition in the UV region and LSPR in the visible region. In contrast, a plasmonic self-powered ultraviolet photodetector that applied rGO/Ag NPs was reported in 2018 [73]. However, this device is based on triboelectric nanogenerators (TENG). A plasmon-assisted photoresponse in rGO/Ag NPs serves as a highly sensitive photoconductive layer. This study showed that the peak absorption at 400 nm light illumination and the absorption level decreased as the wavelength increased to the visible region. Moreover, the influence of the thickness of polydimethylsiloxane (PDMS) towards open circuit voltage (V_OC_) of the device was studied. The self-powered ultraviolet photodetector based on a TENG configuration with the implementation of plasmonic Ag NPs in this work was proposed for the first time.

Based on the previous work, various plasmonic approaches were used in photodetectors. The review mainly focused on Ag- and Au-based NPs [69,72,88]. These noble metal NPs can detect light from the ultraviolet to visible spectrum. In some cases, it can broaden the spectrum of detection up to the near-infrared region. Although both Ag and Au NPs are used in the photodetector, there are reported works that distinguish these materials. Ag is known as a better plasmonic material than Au due to the higher density of plasmon electric fields and narrower energy distribution of generated electron–hole in Ag [87]. This means that Ag can produce higher photoelectric conversion efficiency, which is preferable for self-powering applications.

## 5. Theoretical and Numerical Modelling of MoS_2_/h-BN/Graphene Photodetector

Based on the reviews of materials and plasmonic coupling in Section 3 and Section 4, respectively, it can be summarized that there is room for further improvement in terms of material heterostructures and incorporation of these structures with plasmonic effects, especially for MoS_2_/h-BN/graphene heterostructure that can contribute to the photovoltaic or built-in potential of photodetectors for self-powering applications. Therefore, the non-destructive approach with reasonable timing, cost-efficiency and a large number of samples to mimic the existing device structures for further study and enhancement will be the theoretical modelling and numerical modelling of the device. Computational studies are appealing tools to mimic the chemico-physical properties and behaviors of a system, which results in utmost importance in explaining and foreseeing the system’s behaviors or capabilities. For instance, the modelling work by Zan et al. [55] with density functional theory (DFT) calculation emphasized geometry optimization, because the dimension of each layer contributes towards the electrical properties of the heterostructure. This is supported by the modelling work by Liu et al. that reported the tuning effect of SBH specifically by adjusting the thickness of h-BN in the graphene/h-BN/MoS_2_ design [56]. Currently, the physics and characteristics of these materials or photodetectors/solar cells are being studied through analytical and numerical modelling, such as first principle calculation [40], density functional theory (DFT) [40,89], Quantum ESPRESSO suite [89], Vienna Ab Initio Simulation Package (VASP) [40], molecular dynamics using LAMMPS [89], AFORS-HET [51], 1D-Solar Cell Capacitance Simulator (SCAPS) [46], COMSOL Multiphysics package [90] and Technology Computer-Aided Design (TCAD).

In this section, the potential of numerical modelling using Silvaco TCAD for photodetectors based on MoS_2_/h-BN/graphene heterostructure will be reviewed. Previously, graphene [42,91,92,93], h-BN and MoS_2_ [94] materials were modelled individually or with heterojunctions in Silvaco TCAD, either with ATLAS (device simulator) or ATHENA (process simulator) for devices like transistors, photodetectors and solar cells. With this motivation, a preliminary study with numerical modelling using Silvaco ATLAS on a MoS_2_/h-BN/graphene photodetector, which was adapted from [10], was reported in [5]. This simulation work aimed to replicate the structure in [10] in order to study the physics, behavior and characteristics of photodetectors that employ these materials. The simulation process was conducted by defining meshes, specifying regions based on the structures of the device, defining material properties and boundaries, employing suitable physics models and, finally, solving the modelling by defining the light source and biasing voltages to extract outputs. Important material parameters such as bandgap, electron affinity, permittivity, carrier mobility, work function and complex indices (refractive index (n) and extinction coefficient (k)) were clearly specified to model the materials close to reality.

**Figure 15:**
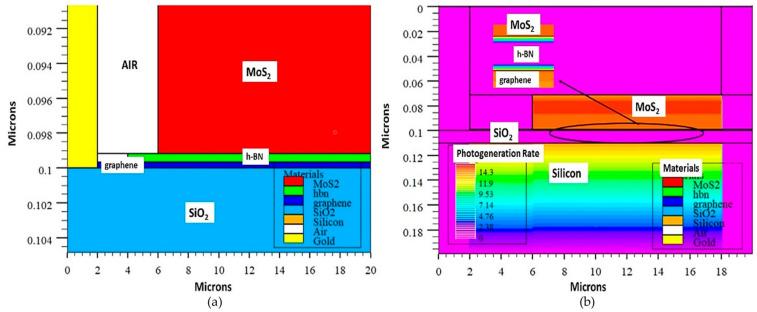
(**a**) Model of MoS_2_/h-BN/graphene photodetector. Adapted from [10] and (**b**) Photogeneration rate in MoS_2_/h-BN/graphene photodetector under illumination at 532 nm (© 2020 IEEE. Reprinted, with permission, from [5]).

To date and to the best of the authors’ knowledge, previous numerical modelling in Silvaco ATLAS highlighted graphene-structured or graphene/MoS_2_-structured devices like photodetectors/solar cells. Therefore, the successful modelling in [5], as shown in Figure 15a, enlightened the path for modelling of graphene, h-BN and MoS_2_ heterostructures at the device level. The photogeneration rate from structural analysis and current density results from this work, as shown in Figure 15b, support the optical and electrical behavior of the fabricated device that was reported in [10]. The highest photogeneration rate was reported in the MoS_2_ layer and gradually decreased at graphene and silicon layers at values of 14.8, 12.9 and 11.4 photons/scm^3^, respectively. This indicates that the modelled MoS_2_ layer had absorbed most of the incident and acted as the absorber, which supports the principle that was highlighted in the device physics. Furthermore, the carrier current density of this model was studied through the cutline at the *x*-axis and showed that electron current density was dominant at the MoS_2_ layer, and hole current density was dominant at the interface of h-BN/graphene, which supports the carrier transport findings as reported in [10]. Further performance improvement by tailoring the dimensions of materials and incorporating plasmonic effects in the device, especially for self-powering applications, can be extended from this framework. Furthermore, the modelling approach can contribute to the fundamental understanding on the effects of plasmonic with respect to MoS_2_/h-BN/graphene heterostructures towards the behavior and performance of photodetectors.

## 6. Conclusions

Self-powering photodetectors convert optical signal to an electrical signal without the need for an external power supply. The performances of the devices are measured based on the performance parameters of photodetectors, which were outlined in Section 2. However, the ultimate issue concerning the self-powering photodetector is the low photocurrent and high dark current at zero bias, which contribute to the low performances of the device. This problem is more significant at low-level optical signals. Therefore, the venture of hybridization of 2D materials and plasmonic coupling approaches are being studied extensively to improve the performance of the self-powering device.

Various types of 2D materials were extensively studied in recent years for photodetection applications. Some specifically focused on implementation of 2D materials for self-powering effects. The most commonly ventured 2D materials to date are graphene and MoS_2_. The combination of superior electrical properties of graphene with the decent optical properties of MoS_2_ make the performance of MoS_2_/graphene hybridized photodetectors much better than the photodetectors with individual graphene or MoS_2_ materials. In this case, MoS_2_ acts as the light sensitizer that absorbs the light, whereas graphene acts as the charge transport layer, which contributes to improved responsivity with higher operation speed of the photodetector. Despite that, suppression of photocurrent and high leakage current at zero bias limits the potential of MoS_2_/graphene photodetectors for self-powering applications. Past studies proposed insertion of a tunnelling layer such as h-BN between these two materials to tackle performance issues in self-powered photodetectors.

On the other hand, metal NPs showed their effectiveness in improving photoresponse by increasing the photon scattering and absorption either by increasing LSPR or contributing to the extraction and transportation of carriers under illumination. Studies also proved that the shape and size of NPs are critical parameters in optimizing the performance contribution of NPs. Plasmonic coupling can be employed to both graphene and MoS_2_ based on previous studies, which demonstrated improved photodetection performances. However, there is room for further research in this area, since the incorporation of hybridized 2D materials such as MoS_2/_h-BN/graphene with plasmonic nanoparticles has not been fully explored. Tailoring the dimensions of this device structure in both vertical and lateral aspects with decorations of plasmonic nanoparticles is necessary for the optimal performance of self-powering photodetectors. The dark current and photocurrent are trade-off parameters with respect to the thickness of the h-BN layer. It is expected that the dark current will be reduced with the increment of h-BN thickness and trade-offs with the photocurrent. This can be countered by improving the absorption in the photodetector with NPs, which can contribute to improved photocurrent.

## Figures and Tables

**Table 1:** Key performance parameters of photodetectors.

Parameters	Descriptions	Equation	Unit
Responsivity (R)	Ratio of photocurrent (*I_ph_*) through the detector to the power of input light, *P_optical_*, where Iph is *I_illuminated_*—*I_dark_*	$\frac{I_{illuminated}-I_{dark}}{P_{optical}}$	A/W
External quantum efficiency (EQE)	Ratio of number of charge carriers collected by the photodetector to the number of incident light. Coefficient of material and thickness of absorbing material influence the EQE.	$\frac{hc}{e\lambda }R$	-
On/off ratio	Ratio of photocurrent towards dark current. It is preferable to have high on/off ratio because it indicates that the device has better photocurrent and lower dark current which contributes to the noise in the device.	$\frac{I_{ph}}{I_{dark}}$	-
Specific detectivity ($D^{*})$	How weak a light the device can detect is defined by the responsivity and dark current of the photodetector. Dark current should be reduced as far as possible as it contributes to the noise in the device and also to distinguish very weak optical signal which can be expressed as follows, where Δf is electrical bandwidth, *i_n_* is noise current and *A* is the effective area of the detector.	$\frac{{\left(A\Delta f\right)}^{1/2}.\;R}{i_{n}}$	Jones
Response speed	Rise time and fall time of the optical signal reaction, which is from 10% to 90% and 90% to 10%, respectively, of the maximum photocurrent	-	s
Operating bandwidth	Frequency at which the output power drops by ½, that is, when photocurrent drops to 70.7%.	-	Hz
Gain (G)	Depends on carrier mobility (µ), photogenerated carriers’ lifetime (τ) and applied field $\left(E\right)$. *L* is channel length of the device.	$\left(µ\tau E\right)/L$	-
Noise equivalent power (NEP)	Required incident power to produce signal-to-noise (SNR) of 1 at 1 Hz bandwidth, which is also given by the noise spectral density, *S_n_*.	$\frac{S_{n}}{R}$	W√Hz
Linear dynamic range (LDR)	Region of incident power within photodetector that has a linear response. It is the logarithm of the ratio between saturation power (*P_sat_*) and NEP.	$10\times \log_{10}\frac{P_{sat}}{NEP}$	dB

**Table 2:** Figures of merit for photodetectors with graphene, MoS_2_ and h-BN heterostructures

Design	Year	Device Fabrication Approach	Dimension (nm)	Operating Condition λ (nm)/V_DS_ (V)	Responsivity (A/W)	On/Off Ratio	Specific Detectivity (Jones)	Ref.
MoS_2_/graphene	2015	G: ME MoS_2_: ME	G: 1L MoS_2_: 3L	520/1.0	$2.06\times 10^{3}$	10^6^	$1.5\times 10^{15}$	[13]
MoS_2_/h-BN/graphene	2016	G: CVD MoS_2_: ME h-BN: ME Dry Transfer	G: 1L MoS_2_: 1L h-BN: 7	405/5	180	10^5^	$2.6\times 10^{13}$	[47]
MoS_2_/h-BN/graphene	2016	CVD	G: 9.32 MoS_2_: 1L h-BN: 20	532/10	$0.3\times 10^{-3}$	6.6	-	[64]
MoS_2_/h-BN/graphene	2019	G: CVD MoS_2_: ME h-BN: ME Dry Transfer	G: 1L MoS_2_: 28 h-BN: 0.47	532/0	0.36	1000	$6.7\times 10^{10}$	[10]
Graphene/MoS_2_/graphene	2019	G: CVD MoS_2_: CVD	Channel length: 15,000 (Lateral Device)	532/1	2	10^6^	10^13^	[63]
Graphene/h-BN/MoS_2_	2020	Dry transfer	G: 1L MoS_2_: 1L h-BN: 10–25	1550/0.02	More than $1\times 10^{8}$	-	-	[30]
Graphene/MoS_2_/graphene	2020	G:CVD Dry transfer	Channel length: 30 (Lateral Device)	432/0.5	$2.2\times 10^{5}$	-	$3.5\times 10^{13}$	[19]

G = graphene, ME = mechanically exfoliated, 1L = monolayer, 3L = tri-layers.

## Data Availability

No new data were created and analyzed in this study.
